# Neuroinflammatory transcriptional programs induced in rhesus pre-frontal cortex white matter during acute SHIV infection

**DOI:** 10.1186/s12974-022-02610-y

**Published:** 2022-10-06

**Authors:** Chase E. Hawes, Sonny R. Elizaldi, Danielle Beckman, Giovanne B. Diniz, Yashavanth Shaan Lakshmanappa, Sean Ott, Blythe P. Durbin-Johnson, Ashok R. Dinasarapu, Andrea Gompers, John H. Morrison, Smita S. Iyer

**Affiliations:** 1 Graduate Group in Immunology, University of California, Davis, CA 95616 USA; 2 Center for Immunology and Infectious Diseases, University of California, Davis, CA 95616 USA; 3 California National Primate Research Center, University of California, Davis, CA 95616 USA; 4 Division of Biostatistics, School of Medicine, University of California, Davis, CA 95616 USA; 5 Department of Human Genetics, Emory University, Atlanta, GA USA; 6 Department of Neurology, School of Medicine, University of California, Davis, CA 95616 USA; 7 Department of Pathology, Microbiology, and Immunology, School of Veterinary Medicine, University of California, Davis, CA 95616 USA

**Keywords:** Rhesus macaque, SHIV, RNA-seq, Neuroinflammation, Neuro AIDS, T cells

## Abstract

**Background:**

Immunosurveillance of the central nervous system (CNS) is vital to resolve infection and injury. However, immune activation within the CNS in the setting of chronic viral infections, such as HIV-1, is strongly linked to progressive neurodegeneration and cognitive decline. Establishment of HIV-1 in the CNS early following infection underscores the need to delineate features of acute CNS immune activation, as these early inflammatory events may mediate neurodegenerative processes. Here, we focused on elucidating molecular programs of neuroinflammation in brain regions based on vulnerability to neuroAIDS and/or neurocognitive decline. To this end, we assessed transcriptional profiles within the subcortical white matter of the pre-frontal cortex (PFCw), as well as synapse dense regions from hippocampus, superior temporal cortex, and caudate nucleus, in rhesus macaques following infection with Simian/Human Immunodeficiency Virus (SHIV.C.CH505).

**Methods:**

We performed RNA extraction and sequenced RNA isolated from 3 mm brain punches. Viral RNA was quantified in the brain and cerebrospinal fluid by RT-qPCR assays targeting SIV Gag. Neuroinflammation was assessed by flow cytometry and multiplex ELISA assays.

**Results:**

RNA sequencing and flow cytometry data demonstrated immune surveillance of the rhesus CNS by innate and adaptive immune cells during homeostasis. Following SHIV infection, viral entry and integration within multiple brain regions demonstrated vulnerabilities of key cognitive and motor function brain regions to HIV-1 during the acute phase of infection. SHIV-induced transcriptional alterations were concentrated to the PFCw and STS with upregulation of gene expression pathways controlling innate and T-cell inflammatory responses. Within the PFCw, gene modules regulating microglial activation and T cell differentiation were induced at 28 days post-SHIV infection, with evidence for stimulation of immune effector programs characteristic of neuroinflammation. Furthermore, enrichment of pathways regulating mitochondrial respiratory capacity, synapse assembly, and oxidative and endoplasmic reticulum stress were observed. These acute neuroinflammatory features were substantiated by increased influx of activated T cells into the CNS.

**Conclusions:**

Our data show pervasive immune surveillance of the rhesus CNS at homeostasis and reveal perturbations of important immune, neuronal, and synaptic pathways within key anatomic regions controlling cognition and motor function during acute HIV infection. These findings provide a valuable framework to understand early molecular features of HIV associated neurodegeneration.

**Supplementary Information:**

The online version contains supplementary material available at 10.1186/s12974-022-02610-y.

## Background

The central nervous system (CNS) houses a complex array of innate and adaptive immune cells facilitating immunosurveillance during homeostasis [1–3]. Furthermore, the CNS is permissive to activated lymphocytes, expressing CNS homing receptors, via the blood–brain and blood–cerebrospinal fluid barriers during the effector phase of the immune response [4, 5]. These features underlie the immune response of neuroinflammation, which evolved to protect CNS tissues. Yet, when sustained, chronic neuroinflammation results in oxidative stress and cellular damage impairing synaptic and neuronal function which underlies the initiation and progression of neurodegeneration [6]. One demographic at a notably higher risk of developing age-related neurodegenerative diseases are people living with HIV (PLWH).

Following mucosal transmission, HIV initially replicates in gut-associated lymphoid tissue with subsequent systemic dissemination and rapid establishment of the viral reservoir in major organ systems, including the CNS. While neurons are not targets for HIV-1, entry of infected immune effectors (CD4 T cells and monocytes) via the barrier systems results in CNS viral dissemination during the acute phase of infection. Antiretroviral therapy (ART) is associated with cognitive improvements; however, 50% of PLWH on therapy remain symptomatic for a spectrum of cognitive impairments called HIV-associated neurocognitive disorders (HAND) [7–10]. A study of nine HIV + PLWH initiating ART within 4 months of acquisition showed evidence for compartmentalized HIV DNA within the cerebrospinal fluid (CSF), indicating establishment of the CNS reservoir early following infection [11]. Viral influx to the CSF was associated with inflammation as evidenced by the association between CSF HIV-1 RNA and CSF neopterin, a marker of inflammation and immune activation [12]. Several studies demonstrate white and gray matter frontal atrophy early following HIV infection [13–15], while neural injury within the striatum and decrease in size of the caudate nucleus are documented in chronic disease [16]. Thus, neuropathological analyses in PLWH demonstrate early CSF viral influx, CNS inflammation and early structural alterations in the frontal cortex as key pathological components of HAND. However, molecular changes underlying acute neuroinflammation in the HIV-infected brain and disruption of cellular programs in the pre-frontal cortex and other regions linked to cognition and motor control remain understudied. Therefore, delineating molecular signatures characteristic of the acute HIV infected brain is important to gain deeper insights into pathways underlying progressive neurodegeneration. Furthermore, RNA sequencing approaches may provide greater resolution into biological processes and networks otherwise overlooked by cellular/tissue-based techniques.

To understand the molecular underpinnings of acute neuroinflammation, we performed RNA-sequencing of the pre-frontal cortex subcortical white matter (PFCw) in animals infected with SHIV.C.CH505 HIV-1 virus (SHIV), a CD4-tropic transmitted/founder (T/F) virus [17, 18]. In addition, three synapse-dense regions; hippocampus (HP), superior temporal sulcus (STS), and caudate nucleus (CN), were chosen based on their relevance to neurocognitive decline and/or known patterns of pathology in neuroAIDS [19–21]. Our approach revealed novel insights into HIV-1-induced transcriptional changes in the brain, and the data support three main conclusions: first, that there is pervasive immune surveillance of the rhesus CNS at homeostasis. Second, HIV-1 dissemination within the brain induces perturbations in key biological pathways regulating inflammation and antiviral signaling, oxidative and nitrosative stress, and endoplasmic reticulum (ER) stress. Third, pathways regulating T helper 1 inflammation and microglial activation are induced within the PFCw during acute HIV-1 infection. Our data provide an important framework to understand determinants of acute neuroinflammation and lay the foundation for identifying immune mechanisms underlying neuropathogenesis in HIV infection.

## Results and discussion

### Study design

To identify major transcriptional features of the brain during acute HIV infection, we reasoned that infecting young animals with a virus that recapitulates properties of T/F viruses would allow us to understand inflammatory consequences of acute HIV-1 dissemination to the CNS in the HIV susceptible demographic. To this end, we infected young adult rhesus macaques (5.2–5.6 [years.months], *n* = 3) intravenously with the R5 CD4 tropic SHIV.C. CH505 virus, a T/F virus with Env substitutions conferring strong engagement with rhesus CD4 resulting in high replication in vivo and consequent depletion of CCR5 + CD4 T cells in mucosal tissues. Furthermore, delayed development of autologous neutralizing antibodies 3–6 month post-infection assures acute viral dissemination [17, 18].

**Fig. 1:**
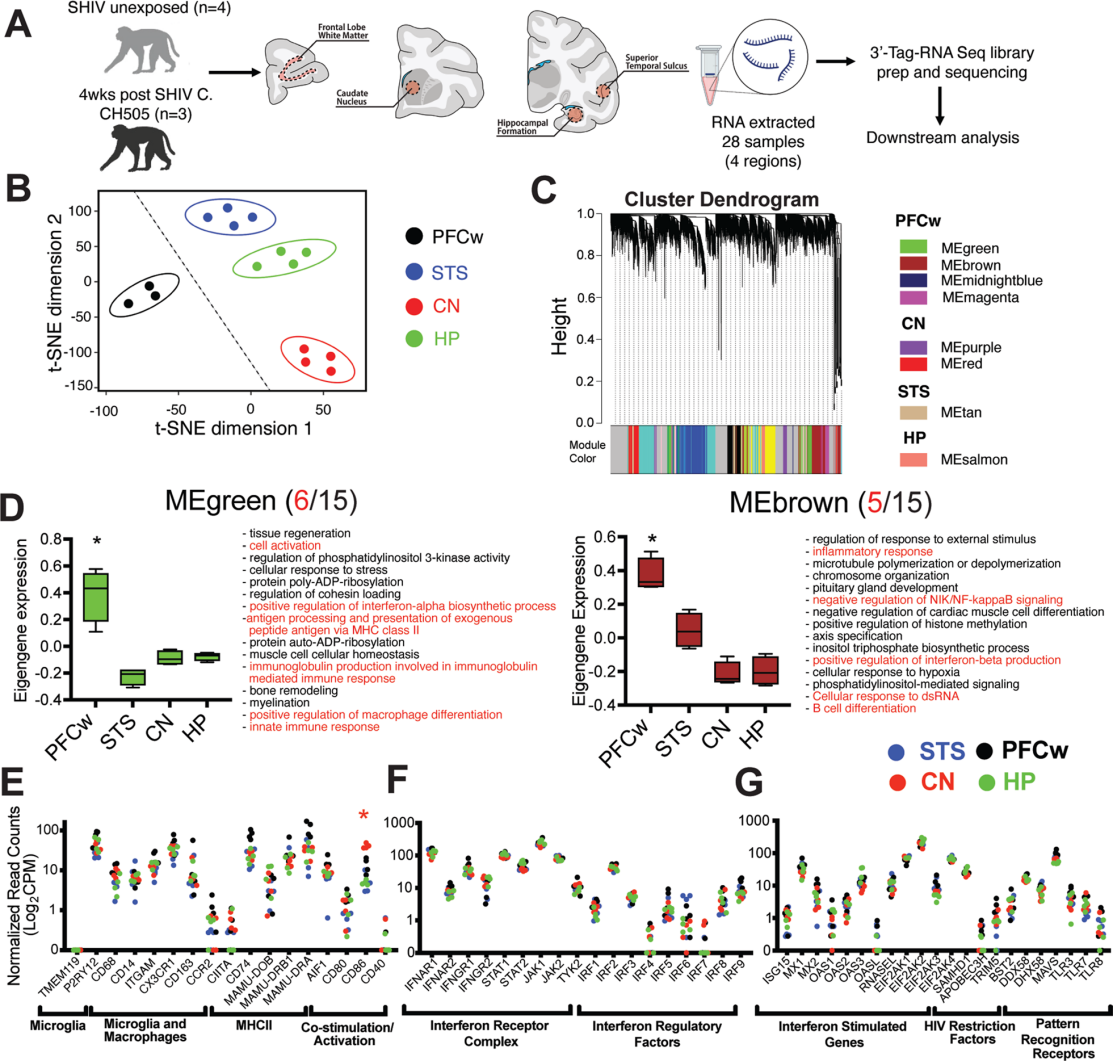
Molecular signatures of innate immune surveillance in the brain of uninfected rhesus macaques. **A** Study schematic: rhesus macaques were infected with SHIV. C.CH505 intravenously (*n* = 3; 3 females; ages 5.2–5.6 [years.months]) and euthanized at 4 week post-infection. Uninfected animals from opportunistic medical culls (*n* = 4; 1 male, 3 females; ages 3.6–6.5 [years.months]) were used as controls. 3 mm brain punches were collected from the subcortical white matter of the prefrontal cortex (PFCw) between Bregma + 16 and + 10 mm, the caudate nucleus (CN) between Bregma + 6 and 0 mm, and the superior temporal sulcus (STS) and hippocampus (HP) between Bregma − 14 and − 20 mm. Total RNA was extracted from bulk tissue samples and 3’-tag RNA-seq was performed. **B** t-Stochastic neighborhood embedding (t-SNE) clustering of gene expression profiles from uninfected control animals. Outlier sample (Animal 43661, pre-frontal cortex white matter control) is removed from the plot. **C** Cluster dendrogram of gene co-expression modules, indicated by color (Left), and region-specific modules (Right) generated from weighted gene co-expression network analysis of tissue-specific gene expression profiles from uninfected animals. **D** Regional eigengene expression and corresponding top fifteen most significantly enriched (*p* < 0.01 by Fisher’s exact test) biological processes GO terms within MEgreen (Left) and MEbrown (Right) modules. Red text indicates terms related to immunological processes. Regional expression levels of genes associated with macrophages/microglia **E** and anti-viral responses **F**, **G** in uninfected animals expressed in Log_2_ counts per million (CPM). **B** Circles indicate 95% confidence intervals. Dotted line indicates separation of white matter and gray matter sample clusters. **D** Box plots indicate quartiles. *P < 0.05 by linear mixed effects models (region effect). **E** * adjusted p < 0.01 and Log_2_ fold change (FC) > 1.5 relative other synapse dense regions (STS, CN, and/or HP). Asterisk color corresponds to a region (CN [red]) for which a gene is differentially regulated relative to other synapse dense regions. **E**–**G** Data points indicate samples from a single brain region and animal

Animals were euthanized at week 4 post-SHIV infection to capture transcriptional perturbations induced by innate and adaptive immune responses to viral seeding in the CNS. At necropsy, the entire brain was excised en bloc, following saline perfusion, and regional punch biopsies were obtained from four major brain regions associated with cognition or motor behaviors. As white matter pathology is commonly observed in Human AIDS, we collected tissue from the subcortical white matter of the prefrontal cortex (PFCw), a region that contains multiple myelinated circuits linked to cognitive and motor functions, to evaluate transcriptional changes in white matter tissue. In addition, we collected samples of synapse-rich areas: the caudate nucleus, a region involved in motor control and typically afflicted in neurotropic HIV infection, in addition to the gray matter of the superior temporal sulcus (STS) and hippocampal formation (HP), which are linked to cognition and memory, respectively, and thus key regions for cognitive decline as seen in aging, AIDS, and Alzheimer’s Disease (Fig. 1A, Additional file 1: Table S1). Brain regions were identified using anatomical maps in the macaque brain atlas [22]. In addition, four SHIV unexposed animals (3.6–6.6 [years.months]) were assessed to establish baseline transcriptome profiles. Following bulk RNA extraction from 28 total samples (SHIV *n* = 12; SHIV unexposed *n* = 16), we performed 3’-tag RNA sequencing on extracted RNA (RNA-seq) from regional tissue punches and obtained gene expression levels, denoted as normalized read counts (NRC).

### Molecular signatures of innate immune surveillance in the rhesus brain

To explore gene expression patterns during homeostasis, we systematically assessed transcriptional features across brain regions in SHIV unexposed animals. Comparison of NRCs demonstrated equivalent gene expression across brain regions (PFCw, 17,717; STS, 17,221; CN, 17,861; HP, 17,550). As illustrated, NRC range (0.139–10,000) and distribution (average 0.5% genes > 1000 NRC, 12.57% genes between 100 and 999.99 NRC, and 87% genes < 99.99 NRC) was comparable across regions (Additional file 1: Fig. S1, Additional file 2: Table S2).

T-distributed stochastic neighbor embedding (t-SNE) analysis of the brain transcriptome revealed regional specification, an observation consistent with topographical gene expression of primate brain transcriptomes [23] (Fig. 1B, Additional file 1: Fig. S2). To identify region-specific biological modules, we used weighted gene co-expression network analysis (WGCNA) and constructed gene co-expression networks [24, 25]. WGCNA of the four brain regions identified a total of 17 modules with 8 region-specific modules denoted by color (PFCw [MEgreen, MEbrown, MEmidnightblue, MEmagenta,], CN [MEpurple, MEred], STS [MEtan], HP [MEsalmon]) (Fig. 1C) demonstrating functional specification by region. Gene Ontology (GO) analysis of regional modules identified functions related to cognitive behavior, sensory processing, synapse formation, neuron projections, metabolic processes, and signaling pathways (Fig. 1D, Additional file 1: Fig. S3). Notably, the PFCw-specific MEgreen and MEbrown modules showed representation of immune functions including innate immune response, positive regulation of macrophage differentiation, antigen processing and presentation, positive regulation of interferon-alpha, cell activation, cellular response to dsRNA, B cell differentiation, negative regulation of NF-kB signaling, and inflammatory response. Enrichment for these terms was consistent with microglia gene signatures in humans and macaques and their increased frequencies in white matter tissue [26, 27] (Fig. 1D). Overall, the data showed spatial regulation of functional processes across the rhesus brain with representation of immunological processes in the white matter.

We next investigated regional expression of genes regulating innate immune responses. While the microglia-specific marker, TMEM119, was not expressed likely due to lower sensitivity of bulk RNA-sequencing in capturing lower expressed transcripts [27], P2RY12, another microglia specific marker was expressed across all regions (Fig. 1E). Markers commonly associated with microglia and macrophages—CD68, CD14, integrin subunit alpha M (ITGAM [CD11b]), CX3CR1 (Fractalkine receptor with corresponding high expression of the ligand, CX3CL1 [Additional file 1: Fig. S4]), and CD163—were also expressed with no regional biases evident. Genes regulating antigen presentation—the major histocompatibility II (MHCII) genes, MAMU–DOB, MAMU–DRB1, MAMU–DRA were expressed across all brain regions. Correspondingly, CIITA, a co-activator regulating MHCII mRNA transcription, was also widely expressed. CD80, a costimulatory ligand for T-cell activation, and allograft inflammatory factor 1 (AIF1), upregulated by activated microglia and referred to as IBA1, were similarly expressed in all regions. CD86, another costimulatory molecule, was enriched in the CN (adjusted *p* < 0.0000001) relative to other synapse dense regions (STS, HP), while CD40 was expressed at low levels (< 1 NRC) across all sampled tissues (Fig. 1E).

Next, we assessed regional expression of genes regulating antiviral responses. The ubiquitously expressed interferon receptor genes (IFNAR1, IFNAR2, IFNGR1, IFNGR2), were found in all brain regions with minimal regional variation (Fig. 1F). Furthermore, genes encoding downstream signal transduction molecules (STAT1, STAT2, JAK1, JAK2, TYK2) were correspondingly expressed as were the interferon regulatory factors (IRF1–9), proteins regulating interferon production (Fig. 1F). We found the interferon-stimulated genes ISG15, MX1, MX2, OAS1–3 and OASL to be widely expressed in parallel with expression of pattern recognition receptors (Fig. 1G), indicating the rhesus brain was poised to respond to viral infection. Expression of HIV restriction factors such as SAMHD1, BST2 (tetherin), TRIM5 (TRIM5α), and the apolipoprotein B mRNA editing enzyme catalytic subunit 3H (APOBEC3H) were also observed in all sampled regions suggesting these tissues may modulate HIV replication efficiency (Fig. 1G). Despite the regional variability in select genes (i.e., CD86), our data indicate that microglia, macrophage, and antiviral gene programs are comparably expressed across the rhesus brain at homeostasis.

### T cell resident immune signatures within the rhesus brain

**Fig. 2:**
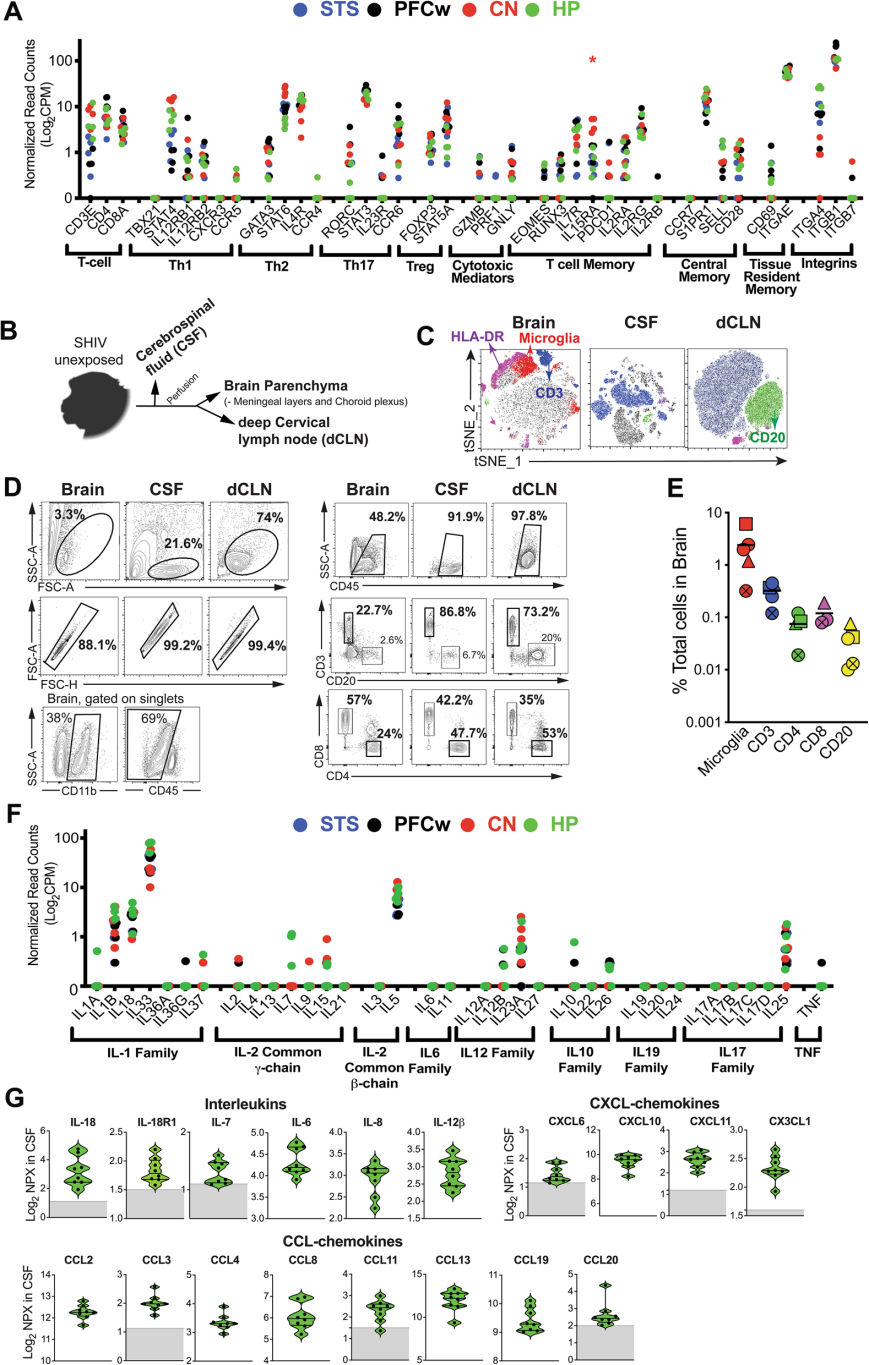
T cell resident immune signatures within the rhesus brain at homeostasis. **A** Regional expression of genes associated with T-cell populations in uninfected animals. Brackets indicate corresponding T-cell subsets associated with genes. **B** Sampling schematic indicating collection of cerebrospinal fluid (CSF), total brain parenchyma and deep cervical lymph node (dCLN) from a comparable group of uninfected rhesus macaques (ages 6 years [two animals], 11 years [one animal], and 16 years [one animal]; one male, three females) for flow cytometric analysis **C–E**. **C** t-SNE plots clustering of immune cell populations in the brain (left), CSF (middle), and dCLN (right) by phenotype. **D** Gating strategy for microglia (CD11b^+^CD45^lo/int^) (Left) and CD4/CD8 T cells (Right) in the brain, CSF, and dCLN. **E** Frequency of microglia and lymphocyte populations among total cells in rhesus macaque brain tissue. **F** Regional expression of cytokine genes in uninfected animals. Brackets indicate cytokine families. **G** Quantification of interleukin, CXCL-chemokines, and CCL-chemokines levels (expressed in Log_2_ Normalized Protein Expression [Log_2_NPX]) in the CSF of a cohort of uninfected animals (age 21–22 years; 8 females) [32]. **A**, **F** Data points indicate samples derived from brain region (indicated by color) and animal. *Adjusted *p* < 0.01 relative other synapse dense regions (STS, CN, and/or HP). Asterisk color corresponds to a region (CN [red]) for which a gene is differentially regulated relative to other synapse dense regions. Data points indicate individual animals **E**, **G,** with symbols indicating specific animals **E**. Bars in violin plots indicate quartiles and gray bars indicate limits of detection (**E**)

We next sought to determine regional expression of adaptive immune genes to understand immunosurveillance of the CNS during homeostasis. Global expression of the T cell chemokines CCL8, CCL24, CXCL12, and CX3CL1 was suggestive of T cell recruitment to both white matter regions, and across three distinct regions of telencephalon, CN, STS, and HP (Additional file 1: Fig. S4). Consistent with this hypothesis, we observed expression of canonical T-cell genes CD3E, CD4, and CD8A across all sampled regions (Fig. 2A). Since STAT proteins both regulate and precede cytokine-induced responses in antigen experienced CD4 T cells, we sought to determine if genes encoding these proteins were concomitantly expressed. STAT4, STAT6, and STAT3 which are pivotal for T_h_1, T_h_2, and T_h_17 CD4 helper programs, respectively, were broadly expressed. We also noted the IL-12 receptor components (IL-12Rβ1 and IL-12Rβ2) which transmit T_h_1 differentiation cues, to be expressed in all regions. CCR5, a T_h_1 chemokine receptor and HIV entry co-receptor, was expressed at low levels (average NRC = 0.09) primarily in the HP (Fig. 2A). In contrast, however, we did not detect expression of the canonical T_h_1 transcription factor, TBX21, and T_h_1 chemokine receptor, CXCR3.

Evaluation of T_h_2 associated genes showed expression of GATA-3 (a T_h_2 differentiation and neurogenesis factor) in all brain regions, except for one HP sample [28]. Furthermore, IL-4R transcripts were also expressed, while expression of the T_h_2 specific chemokine receptor CCR4 was mostly absent (Fig. 2A). The expression of T_h_17-related genes (RORC [RORγt], IL-23R, and CCR6) and Treg associated genes (FOXP3 and STAT5A) (Fig. 2A) was consistent with seeding of the healthy brain by microbiome specific T cells and T regulatory cells [3]. Assessment of cytolytic genes revealed that cytotoxic T cell and NK cell effector molecules (GZMB [granzyme B], PRF1 [perforin 1], and GNLY [granulysin]), which mediate clearance of virally infected cells, were expressed at low levels (NRC < 2). Genes regulating memory CD8 T cell differentiation (EOMES and RUNX3) were expressed in most regions, with the exception for EOMES which was absent in the CN. Cytokine receptors IL-7R and IL-15Rα, necessary for memory T-cell homeostasis, were expressed with significantly elevated levels of IL-15Rα in the CN (adjusted *p* < 0.01) relative to other synapse dense regions, such as the HP and STS. IL-2 receptor family proteins IL2Rγ, which serves as a subunit of the IL-7 and IL-15 receptors, and IL2Rα were also correspondingly expressed. Genes expressed by central memory T-cells (Tcms), CD28 and SELL (CD62L), were observed in all regions, but transcripts for CCR7, a chemokine receptor mediating T-cell trafficking to secondary lymphoid organs, was not detected in any samples. In addition to Tcm markers, signatures associated with tissue resident memory (Trm) T cells such as αE integrin (ITGAE [CD103]) were readily observed in all sampled regions. CD69, another Trm marker, was expressed in the PFCw, STS, and HP, but absent in the CN (Fig. 2A). The sphingosine 1 receptor, S1PR1, mediating T-cell egress was expressed in all three synapse dense regions and PFCw. In addition, we observed widespread expression of integrin beta 1 (ITGB1) across brain regions compared to integrin beta 7 (ITGB7), which is consistent with the role of α_4_β_1_ in regulating immune cell migration to the brain [29]. Together, these data support the prevalence of a heterogenous CNS T cell population composed of T_h_1, T_h_2, T_h_17 with Trm features in the rhesus brain, consistent with T cell profiles reported in healthy mouse and human brains [3, 30].

Next, to substantiate our gene expression data demonstrating surveillance of the rhesus brain by T cells, we performed flow cytometry analysis on CNS tissues from a comparable cohort of SHIV unexposed rhesus macaques. To assess brain-resident lymphocytes, we performed saline perfusion and removed the meningeal layers prior to processing the brain samples. The perfused brain was subjected to collagenase digestion and mononuclear cells were enriched by percoll density gradient (Fig. 2B). Immune cell frequencies in the brain were compared to that in cerebrospinal fluid (CSF) and the CNS draining lymph nodes to delineate relative immune composition in these distinct CNS associated compartments. Consistent with observations in mice and humans, microglia (CD11b + CD45lo/int cells) constituted the predominant immune subset in the rhesus brain (Fig. 2C–E). Furthermore, T-cells were readily observed in the rhesus CNS with brain CD8 and CD4 T cells consisting of approximately 0.1% of total parenchymal cells (Fig. 2C–E).

After establishing CNS surveillance by CD4 and CD8 T cells by flow cytometry, we assessed regional expression of effector cytokine transcripts during homeostasis by RNA sequencing. The IL-1 family cytokines, IL-1β, IL-18, and IL-33 were expressed in all sampled regions (Fig. 2F). Other IL-1 family cytokines along with specific IL-2 common γ-chain cytokines, such as IL-2, IL-9, and IL-15, which regulate immune cell proliferation, were variably detected with relatively low expression levels (< 2 NRC) in the rhesus brain. IL-7, another IL-2 receptor γ-chain family cytokine, was exclusively expressed in the HP, while IL-5, an IL-2 receptor β-chain family cytokine was expressed in all regions. IL-6 and IL-19 family cytokines along with TNF-α, with the exception of one PFCw sample (Animal 44288 [TNF: 0.3 NRC]), were undetectable and expression of IL-12, IL-10, and IL-17 family cytokines was variable among samples. IL-12 family cytokines, IL-12β, a T_h_1 inducing cytokine was expressed in some samples from the PFCw, STS, and HP but was absent in CN. IL-23α subunit that supports T_h_17 differentiation, was expressed in most brain regions. IL-12 family cytokines (IL-12α and IL-27) were not expressed. IL-10 family cytokine expression was variable with IL-10 expressed in the HP and STS of two animals and IL-26 in the PFCw, STS, and HP of three animals (Fig. 2F). Except for IL-25, associated with T_h_2 responses and maintenance of blood brain barrier integrity [31], cytokines of the IL-17 family were not detected (Fig. 2F).

To substantiate homeostatic cytokine and chemokine RNA-seq data, we performed protein-based analysis of the CSF from 8 uninfected rhesus macaques utilizing the O-link platform [32] (Fig. 2G). The cytokine milieu within the CSF is a composite of mediators generated from three distinct compartments; cytokines produced by cells within the brain parenchyma and drained in CSF via the interstitial fluid; cytokines produced by choroid plexus epithelial cells, myeloid cells and T cells within the choroid plexus stroma; and plasma cytokines transported across the blood–CSF (BCSF) barrier [33]. Commensurate with gene expression data, we found the CSF was immunologically rich with expression of IL-1 family cytokines and their receptors (IL-18, IL-18R1); IL-2 common γ chain cytokine, IL-7; innate cytokines, IL-6, and IL-8 and IL-12β subunit. Among the CCL-chemokines, CCL2, produced by choroid plexus epithelial cells and stromal cells [34], was detected as were the CCR5 ligands, CCL3 and CCL4. The T_h_17 chemokine, CCL20 and the T_h_1 chemokines, CXCL10, CXCL11 and CX3CL1 were found in the CSF. In contrast to studies in mice, levels of IL-4 and IFNγ were below the level of detection in macaque CSF (data not shown) [35]. Altogether, these data demonstrate a rich cellular and soluble immune landscape resulting from homeostatic immune surveillance in the rhesus CNS—both parenchymal and CSF compartments.

### Changes in brain transcriptome following SHIV infection

**Fig. 3:**
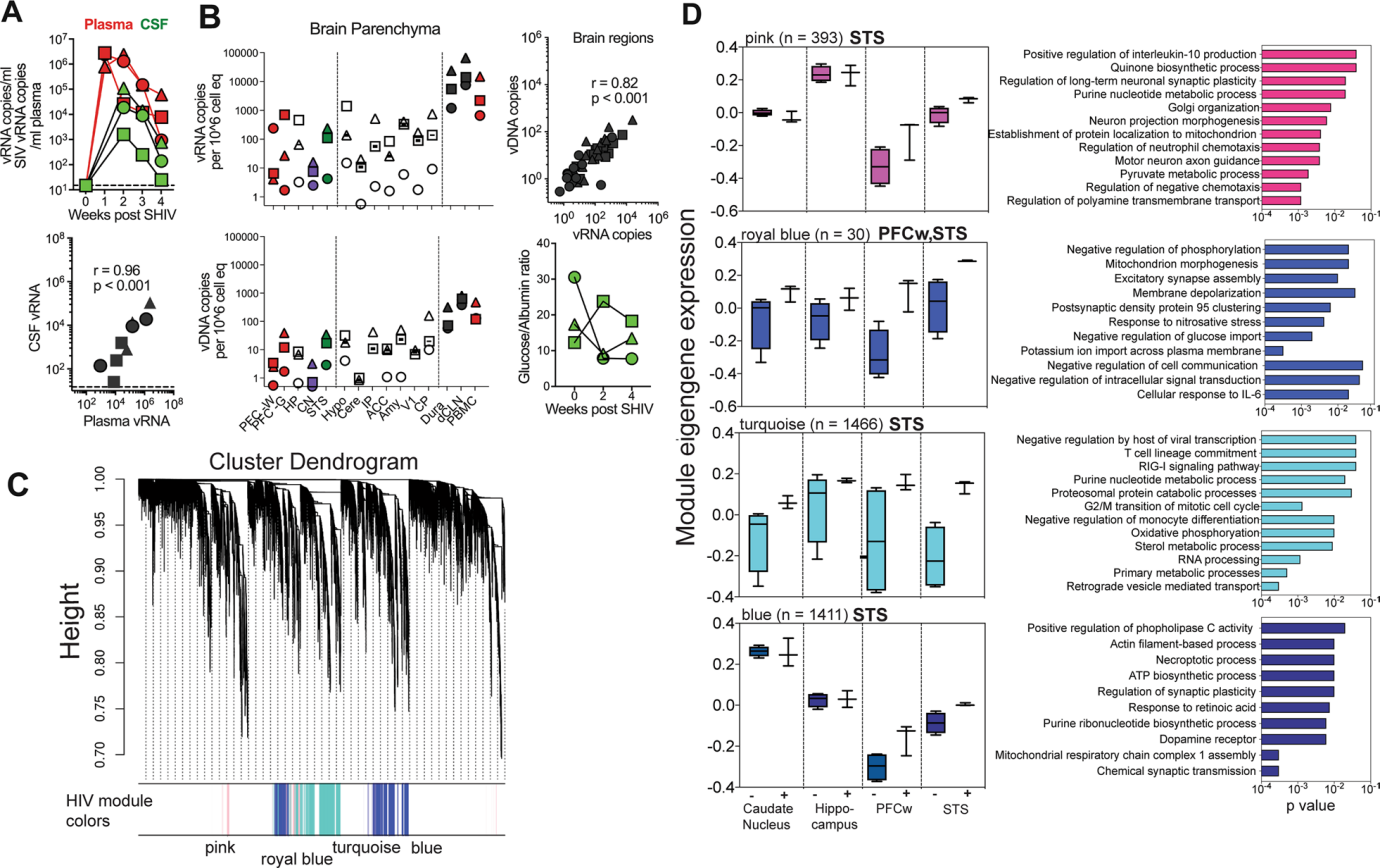
Changes in brain transcriptome following SHIV dissemination in the CNS. **A** Kinetics of plasma (red) and CSF (green) viral loads following intravenous infection with. SHIV.C.CH505. CSF vRNA correlates with Plasma vRNA (*r* = 0.96, *p* < 0.001; two-tailed Spearman correlation). **B** vRNA and vDNA copies in various brain regions, dura mater, deep cervical lymph nodes, and PBMCs. Correlation of vDNA with vRNA from all brain regions sampled (*r* = 0.82, *p* < 0.001; two-tailed Spearman correlation). Bottom graph shows ratio of CSF glucose and albumin (in mg/dL) during acute viremia. **C** Cluster dendrogram showing modules of significantly co-expressed genes associated with SHIV infection indicated by color (pink [393 genes] royal blue [30 genes], turquoise [1466 genes], blue [1411 genes]). **D** Expression levels of module eigengene based on SHIV infection status (+ infected, −uninfected) in the pre-frontal cortex white matter (PFCw), superior temporal sulcus (STS), caudate nucleus, and hippocampus (Left). Significantly enriched biological process gene ontology (GO) terms in SHIV-specific gene co-expression modules (Right). **D** Box plots indicate quartiles. *P* values determined by Fisher’s exact test. **A**, **B** Symbols indicate individual animals. **A** Data points in correlation plot indicate samples collected from individual animals (referenced by shape) and collection timepoint (weeks 1–4). **B** Data points in correlation plot indicate samples collected from individual animals and tissues (Brain regions, Dura mater, deep cervical lymph nodes, and PBMCs).

After establishing molecular features of the adult macaque brain, we sought to delineate changes in the brain transcriptome during acute SHIV infection. To determine CNS viral dissemination, we first assessed CSF vRNA trajectories during acute viremia (Fig. 3A). Viral kinetics in cell-free CSF paralleled plasma viremia, with 2 log fold lower viral loads in the CSF compared to plasma (week 2 median viral loads/ml: plasma, 1.3 × 10^6; CSF, 19,000), with a strong correlation between these distinct compartments (*r* = 0.96, *p* < 0.001) (Fig. 3A). To formally assess viral dissemination within the rhesus CNS, we performed a comprehensive assessment of vRNA and vDNA across several brain regions and CNS tissues obtained post-mortem. The data showed dissemination of SHIV (vRNA, expressed as copies/10^6 cell equivalent) to regions sampled for RNA sequencing analysis: the PFC white matter (4.1–240), HP (3.3–460), STS (4.3–240), and CN (2.5–16). Furthermore, we observed detectable vRNA in the PFC grey matter, Hypothalamus (Hypo), Cerebellum (Cere), Inferior Parietal (IP), Anterior Cingulate Cortex (ACC), Amygdala (Amy), and primary visual cortex (V1) of the brain, and the choroid plexus stroma (CP) indicating widespread dissemination of SHIV within the rhesus CNS (Fig. 3B). The presence of vRNA in the Dura mater (1200–24,000) and deep cervical nodes (7700–66,000) was consistent with CNS antigen clearance via the lymphatics. vRNA strongly correlated with pro-viral DNA levels (r = 0.82, p < 0.001), suggesting early seeding of viral reservoirs in major brain regions (Fig. 3B), consistent with reports that blocking T cell and monocyte extravasation via the blood–brain barrier significantly decreases vDNA in the frontal cortex in SIV infected rhesus macaques [29]. The stability of the CSF Glucose/Albumin ratio indicated that CNS viral entry was not associated with breakdown of the blood–brain or the BCSF barriers, suggesting immune cell recruitment to the CNS may mediate viral entry (Fig. 3B).

To identify principal features of the SHIV infected brain, we conducted WGCNA on all 28 brain samples from both infected and uninfected animals using log_2_ counts per million reads (Fig. 3C). We identified a total of 21 gene co-expression modules with 4 modules highly associated with SHIV infection: pink (393 genes), turquoise (1466 genes), and blue (1411 genes) within the STS, and royal blue (30 genes) in the PFCw and the STS. Figure 3D shows module eigengenes characterized by their expression patterns in different brain regions and the corresponding GO terms enriched in each module.

Genes within these 4 modules were enriched for GO terms corresponding to neuroinflammation, such as oxidative and nitrosative stress (e.g., microsomal glutathione S-transferase 3 [MGST3], peroxiredoxin [PRDX3], and the mitochondrial citrate carrier, SLC25A43) (Additional file 1: Fig. S5). While we did not observe differential expression of genes regulating excitotoxicity, several genes regulating synaptic markers were differentially induced, e.g., the synaptobrevins (vesicle associated membrane protein [VAMP] 3, 8), synaptophysin, and dynamin 3; suggestive of early synaptic changes following SHIV infection **(S5)**. Despite the low abundance of synapses in white matter tissues, alterations in synaptic gene pathways observed in the PFCw could be attributed to interstitial white matter neurons reported in both human and rhesus white matter tissue [36, 37]. Altogether, these data show viral entry and dissemination within the CNS, with corresponding upregulation of gene programs associated with neuroinflammation in the PFCw and STS.

### Enrichment of inflammatory gene signatures in the PFC white matter following SHIV infection

**Fig. 4:**
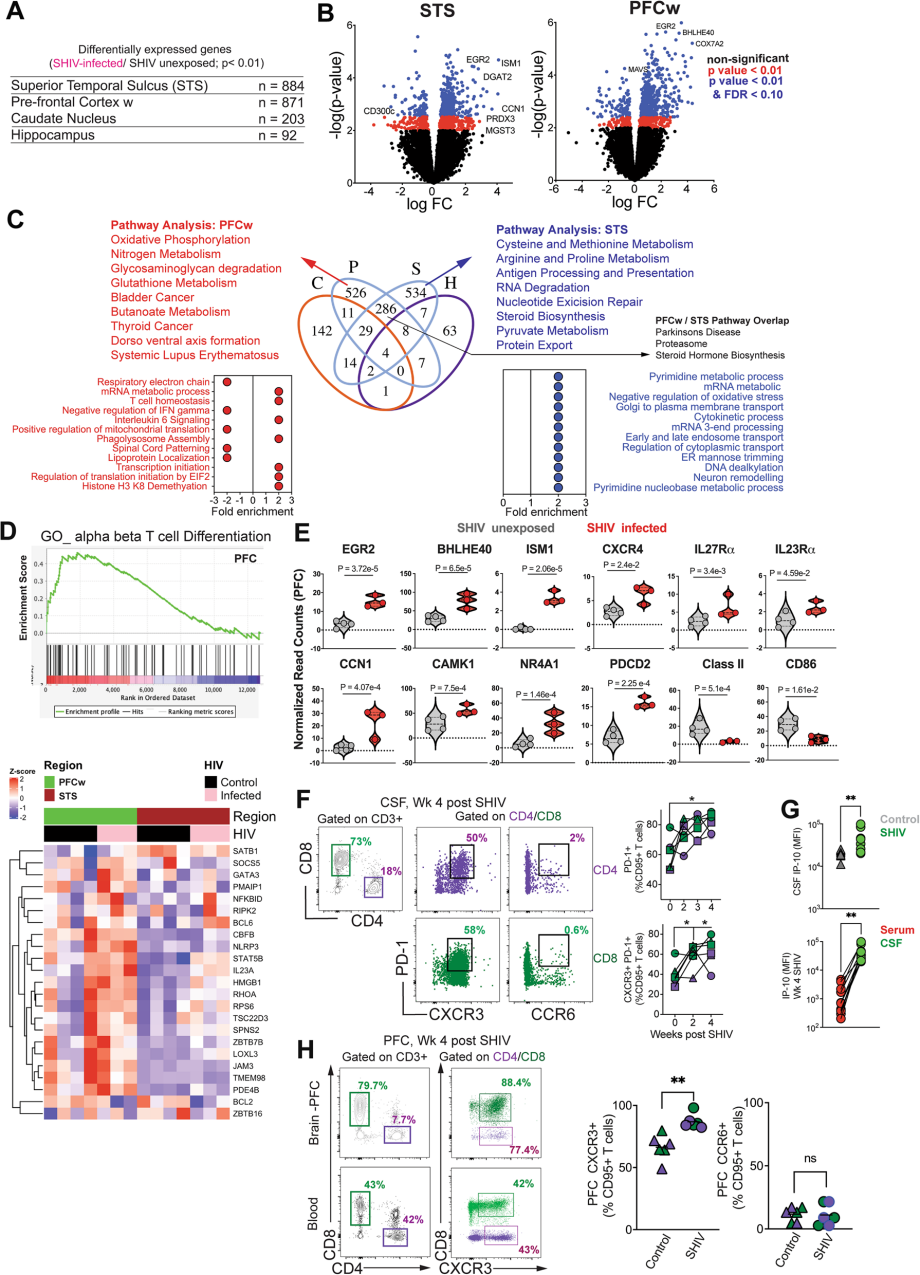
SHIV-induced alterations of genes regulating inflammatory pathways in the PFC white matter. Differential gene expression analysis between SHIV infected and unexposed animals. **A** Number of differentially expressed genes (DEGs) (adjusted p < 0.01) with respect to SHIV infection status identified in the STS, PFC white matter (w), CN, and HP. **B** Volcano plots displaying Log fold change (FC) and significance (-log *p* value) of genes expressed in the STS (Left) and PFCw (Right) after SHIV infection. Colors indicate significant thresholds of genes (*p* value < 0.01 and False discovery rate (FDR) adjusted *p* value < 0.10 [blue]; *p* value < 0.01 [red]; non-significant [black]). **C** Venn diagram indicating the number of overlapping DEGs from SHIV infected versus uninfected comparisons in the PFCw (P), STS (S), CN (C), and HP (H) (Center). GSEA–KEGG pathway analysis of DEGs exclusive to the PFCw (Left, Red), STS (Right, blue), or shared between the PFCw and STS (Right, gray). Plots indicate fold change by GSEA–GP–BP pathway enrichment between SHIV infected and uninfected animals in the PFCw (Left, Red) and STS (Right, Blue). **D** Gene set enrichment analysis of transcripts related to alpha beta T cell differentiation GO:0046632 from PFCw DEG profiles (Top) and heatmap showing expression levels of corresponding genes in the PFCw (green) and STS (red) of SHIV infected (pink) and uninfected (black) animals (Bottom). **E** Expression level of DEGs regulating inflammatory processes, immune cell functions, and synaptic functions in the PFCw of SHIV infected (red) and uninfected (gray) animals.** F** Gating for activated CD4 T-cells (top) and CD8 T cells (bottom) and frequency of PD1 + , PD1^+^CXCR3^+^, PD1^+^CCR6^+^ CD4 T-cells in the CSF of SHIV infected and uninfected animals over the course of infection. **G** IP-10 levels in CSF and serum expressed as Median Fluorescence Intensity (MFI) run using Legend plex assay in controls and SHIV infected animals (*n* = 7 animals infected with SHIV.C.CH505 intravaginally and *n* = 3 intravenously). **H** Gating for activated T cells isolated from the PFC**,** frequency of CXCR3 + CD4 and CD8 T cells in controls versus SHIV infected animals**.** Symbols indicate individual animals. **A**, **B, F–H** *P* values for gene expression data derived from linear mixed effects model. **F**,** H** *P < 0.05 by Wilcoxon sign ranked test. **G** **P < 0.01 by Mann–Whitney test

To investigate gene expression changes resulting from CNS viral dissemination, we first applied t-SNE analysis to gene expression data from all samples, which revealed clustering of gene expression profiles between infected and control animals by brain region (Additional file 1: Fig. S6). Using DEG analysis to compare infected versus uninfected animals (criteria: *p* < 0.01 threshold for false discovery rate [FDR] and absolute value of log2 fold change ≥ 1), we found regional differences in SHIV-induced transcriptomic changes (STS: 884 DEGs; PFCw: 871 DEGs; CN: 203 DEGs; HP 92 DEGs) (Fig. 4A) with the STS and PFCw exhibiting the most distinctive transcriptional profiles following infection. Notably, differentially expressed genes in PFCw and STS were EGR2 and BHLHE40, transcription factors regulating IFNγ production from CD4 T cells and microglia [38, 39] and ISM1 secreted by activated CD4 T cells [40]. COX7A2 and DGAT2 involved in oxidative phosphorylation and lipid metabolism, respectively, were also induced implicating these pathways in the acute neuroinflammatory response to SHIV infection [41, 42]  (Fig. 4B, Additional File 1: Fig S5).

To gain insights into molecular programs induced following SHIV infection, we conducted pathway analyses for DEGs associated with SHIV infection in the PFCw and STS. Of the 526 uniquely regulated genes in the PFCw, pathways regulating oxidative phosphorylation, glutathione metabolism, and Systemic Lupus Erythematosus were enriched alongside processes encompassing T cell homeostasis, and IL-6 signaling. Within the STS, amino acid metabolism, antigen processing and presentation, and steroid biosynthesis pathways were most significantly upregulated after infection. The 286 DEGs that overlapped between the PFCw and STS represented pathways associated with Parkinson’s Disease, the proteasome pathway, and steroid hormone biosynthesis (Fig. 4C). These data reveal perturbation in important immune, neuronal, synaptic, and metabolic pathways within key anatomic regions controlling cognition during acute SHIV infection.

Next, we sought to identify genes regulating inflammatory processes within the PFCw. To this end, we integrated expression analysis with inflammatory biological pathways utilizing gene set enrichment analysis (GSEA). GSEA revealed enrichment of genes regulating T cell differentiation (Fig. 4D); these included T cell chemotaxis (enrichment score [ES] = 0.65; top genes PIK3CD, STK39, PIK3CG, WNK1, CCR2 and CXCL16); T cell differentiation (ES = 0.5; top genes, RARα, IL23α, LEF1, GATA3, NLRP3, IRF1, BATF, and BCL-6), (ES = 0.5), and response to IL-6 (ES = 0.45; top genes, STAT3, RELA, SMAD4, CBL, IL6R, IL6ST, JAK1, ST3GAL6).

Several genes among those differentially expressed in the PFCw encoded for proteins regulating T cell and microglial activation. Among the most highly induced genes was the transcriptional regulator, EGR2, which has been implicated in neuronal plasticity in response to inflammation and T_h_1 CD4 T cell proliferation [38, 43]. Similarly, the IL-1β-induced transcription factor, BHLHE40, which negatively regulates IL-10 production and drives T cell mediated neuroinflammation was also upregulated in the PFCw following infection [39, 44]. ISM1, a gene encoding the secreted protein Isthmin which regulates NK and T_h_17 CD4 T cell effector functions [40], was also induced (Fig. 4E).

Several genes related to chemokine and cytokine receptors showed elevated expression levels within the PFCw following SHIV infection. This included CXCR4, a chemokine receptor, which is elevated in neurodegenerative diseases and expressed by a variety of immune cells, including T cells, and activated microglia [45]. Cytokine receptor subunits IL-27Rα and IL-23Rα, expressed by activated immune cells were significantly induced in the PFCw after infection [46, 47]. Connective tissue growth factor/Nephroblastoma overexpressed gene family 1 (CCN1), an inducible extracellular matrix protein secreted by reactive astrocytes was upregulated in the PFCw [48]. The kinase, CAMK1, which regulates CCN1 expression and is induced in astrocytes following Zika infection [49], was also correspondingly elevated (Fig. 4E). This gene expression program indicated that astrocyte, microglial, and T cell activation programs were induced in the frontal cortex white matter following SHIV infection. In contrast to genes regulating immune cell activation, we also found that the orphan nuclear receptor and transcription factor NR4A1 (NUR77), an intrinsic negative regulator of microglial and T cell activation [50], was upregulated. Similarly, the immune checkpoint inhibitor, programmed cell death-2 (PDCD2) was also elevated in the PFCw. Intriguingly, expression of HLA-DP and CD86 were decreased, suggestive of a net diminution in expression or reduction in numbers of antigen presenting cells in the PFCw following infection (Fig. 4E).

Based on these transcriptional changes and SHIV dissemination within distinct brain regions, we hypothesized that activated T cells were actively recruited to the CNS during acute SHIV infection. To this end, we assessed immune composition of the CSF over the course of infection and found increased frequencies of CD4 and CD8 T cells expressing PD-1, a marker for TCR stimulation, indicative of CNS influx of activated T cells during acute infection. We observed a significant increase in CXCR3 + PD-1 + CD4 and CD8 T cells denoting potential recruitment of antigen-specific cells (Fig. 4F), consistent with reports of CD8 T cell influx into the CSF during acute HIV-1 infection [51]. However, unlike the reported influx of T_h_17 in neuroinflammatory disease models, negligible expression of CCR6 was found on CSF CD4 T cells. This increase in CXCR3 + CD4 and CD8 T cells was consistent with induction of IP-10 in CSF following infection (Fig. 4G), and the relative higher levels of IP-10 in CSF compared to the systemic compartment, indicating CNS production of IP-10 both during homeostasis and infection. In line with the increase in T_h_1 inflammatory response in the CSF, we found evidence for a net increase in CXCR3 + CD4 and CD8 T cells in the PFC during acute SHIV infection (Fig. 4H). Together, these data demonstrate that viral dissemination together with T_h_1 CD4 T cell and CXCR3 + CD8 T cell infiltration within the CNS occur during acute stages of infection and correspond with the induction neuroinflammatory gene programs in the PFCw.

In addition to immune genes, we found that the primary astrocyte GABA/glycine transporter SLC6A11, within the neurotransmitter family, was upregulated in the SHIV infected PFCw relative to uninfected controls. While SLC6A11 is not altered in neurodegenerative diseases, a net increase in reactive astroglia could explain increased levels of this transporter [52] (Additional file 1: Fig. S7). SLC32A1, which mediates GABA/glycine uptake into synaptic vesicles was also upregulated. Correspondingly, we observed increased expression of the mesencephalic astrocyte-derived neurotrophic growth factor (MANF)—an ER stress inducible factor reported to rescue neuronal loss in several neurological disorders [53]. In addition, genes regulating RNA levels of ATP synthase and transport subunits were induced after SHIV infection suggestive of alterations in mitochondrial respiratory capacity and cholesterol synthesis. These observations suggest that molecular changes in synaptic functions and bioenergetic pathways in the brain can occur during the early stages of SHIV infection.

Rhesus macaque models have been informative in characterizing SIV associated neuroinflammatory processes, with models of neurotropic SIV infection showing increased frequencies of memory CD4 T-cells, B-cells, and macrophages in the brain parenchyma of infected animals developing encephalitis versus animals without encephalitis [54]. Studies using SHIV have demonstrated early viral seeding of the CNS and CD4 T cell infiltration into the brain parenchyma using immunohistochemistry, suggesting that neuroinflammatory processes may be initiated during the early stages of infection [55]. Our findings add to this important body of work by demonstrating features of SHIV-induced transcriptional perturbations during the acute phase of infection within regions of the brain associated with cognition, memory, and motor control. Interestingly, the neocortical association regions STS and the prefrontal subcortical white matter appeared to be more vulnerable to virus-induced alterations than caudate and hippocampus, which mirrors their enhanced vulnerability to age-related changes. This may be attributed to the magnitude of inflammatory processes resultant from initial viral dissemination within the PFC and STS, as observed viral loads were not markedly higher in these regions. These data emphasize the need for deeper analysis into the composition and activation status of immune cells within spatially distinct regions of the brain following HIV infection to identify key cellular players in HIV associated neuroinflammation. In all, our findings demonstrate vulnerabilities within these key regions both to HIV-1 dissemination and neuroinflammation during the acute phase of infection.

While our reported observations are novel, our study has several limitations. First, we performed our analysis in young adult rhesus macaques in consideration of age range of populations vulnerable to primary HIV infection; therefore, whether the extent of neuroinflammatory shifts following HIV-1 infection differ across the age spectrum and during chronic HIV infection are important questions that were not addressed in this study. Second, given observed viral influx in both the white and synapse-rich areas of the PFC, transcriptional changes to gray matter areas within the PFC warrant consideration. Third, our extensive virological analysis demonstrated viral influx and evidence for integration within multiple brain regions, including the hypothalamus, amygdala, and cerebellum. Capturing molecular changes in these regions and using single cell approaches to gain comprehensive insights into pathways disrupted within neuronal, glial, and immune cells during acute HIV infection is an important consideration for future studies. Finally, histopathological assessments of the PFC and STS, not performed herein, would provide important insights into whether observed transcriptional perturbations were associated with pathological sequalae during the acute HIV infection.

In summary, our transcriptomic data, flow cytometric analysis of immune cell populations and quantification of CNS cytokines reveal pervasive immune surveillance of the rhesus CNS at homeostasis. Comparison of transcriptional profiles between uninfected animals and SHIV infected animals revealed significant alterations to gene expression in the PFCw and STS after infection. These changes included differential expression of genes associated with IL-6 responses, oxidative stress, and T-cell differentiation, activation, and chemotaxis. Furthermore, we observed SHIV-associated alterations in genes regulating astrocyte homeostatic functions, synaptic functions, and bioenergetic pathways suggesting that neuroinflammatory processes may alter normal neuron and glial cell activity in the brain. These neuroinflammatory gene profiles were substantiated by flow cytometric analysis which showed influx of T_h_1 CD4 T and CXCR3 + CD8 T cells into the CNS of infected animals over the course of infection. Together the data demonstrate that neuroinflammatory changes to the CNS, within the first weeks following SHIV infection, drives early functional molecular changes in normal neuronal and glial cell populations. Overall, our data provide hitherto unappreciated insights into the impact of SHIV infection on molecular processes in the rhesus brain; the data will be a useful primer to understand molecular mechanisms that drive subsequent neurodegeneration.

## Methods

### Rhesus macaques

Seven colony bred Rhesus macaques (*Macaca mulatta*, 1 male, 6 females, age 3.6–6.6 [years.months]) housed at the California National Primate Research Center (CNPRC) in accordance with the American Association for Accreditation of Laboratory Animal Care guidelines were used for the study. All protocols used in the study were approved by the Institutional Animal Care and Use Committee at UC Davis. Animals in the SHIV infected group were previously immunized with Clade C DNA-prime/protein vaccine and were infected 20 weeks after final protein boost through the intravenous route with SHIV. C.CH505, as described previously[56]. Infected animals were euthanized at 4 week post-infection. Viral titers were assessed in the blood and cerebrospinal fluid (CSF) of SHIV infected animals on a weekly basis. For blood and CSF collections, animals were anesthetized with 10 mg/kg ketamine administered intramuscularly. EDTA-anticoagulated blood was collected via intravenous blood draws and CSF by cisterna magna puncture. SHIV unexposed control animals were selected from opportunistic aged-matched medical culls for unrelated non-neurological conditions and showed no history of neurological impairment (Additional file 1: Table S1).

### SHIV. C.CH505 virus and preparation

SHIV.C. CH505 was selected as the virus recapitulates attributes of primary HIV-1 strains. Briefly, SHIV.C. CH505 is a T/F virus engineered to strongly bind rhesus CD4 T cells while retaining antigenicity and tier2 neutralization sensitivity of the ancestral HIV-1 C.CH505 [17]. Replication efficiencies of SHIV.C. CH505 are comparable to pathogenic SIV strains in vivo and result in depletion of CCR5 + CCR6 + CD4 T cells within mucosal tissues [18]. SHIV. C.CH505 virus stocks at 189 ng/mL propagated in primary activated rhesus CD4 T cells were provided by George Shaw and Nancy Miller (National Institute of Health) and stored in liquid nitrogen. On day of virus administration, stocks were thawed on ice water and diluted fourfold in RPMI 1640. 0.5 mL of working inoculum was loaded into 1 mL Tuberculin syringes and stored on ice until animal challenge.

### Brain tissue collection and RNA extraction

At necropsy, brains were perfused in-vivo via cardiac saline perfusion  and removed from the cranial cavity en bloc. Four regions were chosen based on their relevance to neurocognitive decline and/or known patterns of pathology in neuroAIDS. We were also informed by our preliminary microscopic observations in SIV-infected rhesus monkeys (data not shown). Subcortical white matter associated with the dorsolateral pre-frontal cortex was chosen due to the age-related myelin pathology described for this region that drives cognitive decline in aged monkeys[57] as well as the occurrence of similar white matter pathology observed in neuroAIDS cases [13, 58, 59]. The hippocampal formation (HP) was chosen for its relevance to age-related cognitive decline and neurodegenerative disorders in general [19]. Superior temporal sulcus gray matter (STS) was chosen for similar relevance to cognitive function and the high degree of microscopic pathology seen in preliminary analyses of rhesus macaques following SIV infection (data not shown). Finally, the caudate nucleus (CN) was included due to the extensive pathology seen in this motor system structure in neuroAIDS [20]. Tissues for RNA-seq were collected as follows: PFCw—a scalpel was used to dissect the white matter of the medial and inferior frontal gyri, between Bregma + 16 and + 10 mm, corresponding predominantly to the white matter subjacent to the dorsolateral prefrontal cortex. Care was exercised to minimize the collection of gray matter; CN—a round punch (3 mm in diameter) was collected from the head of the caudate nucleus, between Bregma + 6 and 0 mm, with negligible involvement of the internal capsule; HP—3 mm round punch was collected between Bregma -14 to -20 mm and involved all major subdivisions of the dentate gyrus and hippocampus proper, as well as part of the adjacent subicular complex while excluding the entorhinal cortex; STS—at approximately the same level of the hippocampal formation biopsy, a 3 mm round punch was taken from the intermediate portion of the superior temporal sulcus, involving both the dorsal and ventral banks. The punch was performed at an angle with respect to the surface of the block, allowing the experimenter to follow the progressively dorsal position of the STS in the rostrocaudal direction, which minimized the inadvertent collection of neighboring white matter. Tissue samples collected from the PFCw, STS, CN, and HP were stored in RLT buffer with ß-mercaptoethanol (BME), flash frozen, and stored at − 80 °C until RNA extraction. RNA was extracted from samples using a Qiagen RNeasy lipid tissue mini kit and sent to the UC Davis DNA Technologies and Expression Analysis Core for 3’-tag RNA sequencing.

Additional samples were collected from the deep cervical lymph nodes (dCLN), choroid plexus stroma (CP), dura mater (dura), prefrontal cortex gray matter (PFCg), hypothalamus (Hypo), cerebellum (Cere), inferior parietal (IP), anterior cingulate cortex (ACC), amygdala (Amy), and visual cortex (V1) of the brain and used exclusively for viral RNA and DNA quantification. Brain tissues from these regions were obtained as follows: PFCg—3 mm punches were obtained from both dorsal and ventral banks of the dorsolateral prefrontal cortex, along the principal sulcus, corresponding to Brodmann area 46; Hypo—a scalpel was used to isolate a block containing the anterior and tuberal divisions of the hypothalamus. The block was dorsally limited by the anterior commissure and laterally confined by the internal capsule; Cere—a scalpel was used to dissect a small sample of the lateral hemisphere of the posterior cerebellar lobe. The resulting sample consisted almost exclusively of cerebellar cortical matter; IP—a scalpel was used to isolate the gray matter on the surface of the inferior parietal lobule, corresponding to Brodmann area 7; ACC—3 mm punches were obtained from the gray matter of the supracallosal anterior cingulate gyrus, corresponding to Brodmann area 24; Amy—3 mm punches were collected from the basolateral complex and the medial and central nuclei of the amygdala; V1—3 mm punches were collected from Brodmann area 17, along the calcarine sulcus. Care was taken to minimize the inadvertent collection of white matter; CP—choroid plexus samples were extracted from the temporal and occipital horns of the lateral ventricle.

### 3’-Tag RNA sequencing

RNA integrity and quality was confirmed using microcapillary electrophoresis on an Agilent Bioanalyzer. Libraries were sequenced using 3’tag-RNA-seq on an Illumina HiSeq 4000 platform as previously described [63. ] with slight modifications. In brief, barcoded sequencing libraries were prepared each from 500 ng total RNA sample and libraries' fragment size distribution were verified using micro-capillary gel electrophoresis on a LabChip GX system (PerkinElmer, Waltham, MA). Libraries were quantified using a Qubit fluorometer (LifeTechnologies, Carlsbad, CA), and pooled into equimolar ratios. The library pool was Exonuclease VII (NEB, Ipswich, MA) treated, SPRI-bead purified with KapaPure beads (Kapa Biosystems/Roche, Basel, Switzerland), and quantified via qPCR with a Kapa Library Quant kit (Kapa Biosystems) on a QuantStudio 5 RT-PCR system (Applied Biosystems, Foster City, CA). Libraries were sequenced on a HiSeq 4000 sequencer (Illumina, San Diego, CA) with single-end 100 bp reads with 12 million raw reads per sample. The raw read data were filtered using HTStream (version 1.3.2) which included screening for contaminants (such as PhiX), removal of PCR duplicated reads, rRNA removal, quality-based trimming, and adapter trimming. STAR (version 2.7.9a) [61] was used to align the processed data to the rhesus genome (Mmul10). Custom R code was then used for read and alignment quality assessment as well as to collate counts into a single table for downstream analysis.

### Differential gene expression analysis

Genes were excluded from differential gene expression analysis if they were expressed in less than 50% of samples for at least one brain region, lacked annotation, or were found to have duplicated gene symbols. Differential gene expression analysis was performed using the limma-voom Bioconductor pipeline (limma version 3.44.3, edgeR version 3.30.3) in R. Analysis in limma used a model including effects for brain regions, SHIV infection, and their interactions. Estimates and standards errors were adjusted for within-animal correlations. Differentially expressed genes (DEGs) were defined as genes with *p* value < 0.01 and adjusted (False discovery rate correction) *p* values < 0.1 unless otherwise indicated. Gene ontology (GO) and KEGG pathway enrichment analysis was performed using ShinyGO v0.60 webserver [62].

### Weighted gene correlation network analysis (WGCNA)

The weighted gene co-expression network analysis (WGCNA) was implemented using the WGCNA R package, version 1.69, in R version 4.0.1. WGCNA was performed using a signed network with a robust biweight midcorrelation. A soft-thresholding power of 27 was chosen for analysis of data from uninfected control animals and 23 for analysis of data from both uninfected control and SHIV exposed animals. Soft-thresholding power was set using the pickSoftThreshold function, to achieve a scale-free topology index greater than 0.85. Modules associated with a single brain region were identified by fitting a linear fixed effects model to each module eigengene. A module was defined as being associated with a single region if the eigengene was significantly higher in that region compared to all other regions, based on the region effect from the linear mixed effects model. Similarly, modules significantly associated with SHIV infection were defined as such if the module eigengene was significantly higher in SHIV infected samples compared to uninfected control samples for at least one brain region. The linear mixed effects model for both analyses utilized a fixed effect for brain region and a random effect for animals, and residual variance were allowed to vary by brain regions. For the analysis of samples from both uninfected control and SHIV exposed animals, SHIV infection was also incorporated as a fixed effect in the linear mixed effects model. Gene ontology (GO) enrichment analyses were conducted using Fisher's exact test as implemented in the Bioconductor package topGO. Results reported are for the biological process (BP) GO terms.

### Inflammatory analytes

For measurement of inflammatory analytes, triton inactivated samples were shipped to Olink Proteomics at Stanford University. A 92-biomarker inflammation panel was run according to manufacturer’s instructions. Protein levels are expressed as normalized protein expression (NPX) values, an arbitrary unit. Inflammatory analytes in CSF and plasma were analyzed using a proximity extension assay (O link, Proteomics) as described [63].

### Flow cytometry

Cell staining was performed as previously described [32]. Briefly, surface staining was performed with the following antibody panel (Additional file 1: Table S3) for 30 min at 4 °C. After washing with 1X phosphate buffered saline (PBS), cells were fixed and permeabilized using Cytofix/Cytoperm buffer set for 20 min at room temperature in the dark. Cells were washed twice with 1X FACS buffer and re-suspended in 200μL of FACS buffer. Fluorescence was measured using a BD FACSymphony cell analyzer with FACSDiva version 8.0.1 software (FLowJo LLC). Compensation, gating, and analysis were performed using FlowJo (versions 9 and 10). Reagents used for flow cytometry are listed in Additional file 1: Table S3.

### Quantitative RT-PCR for viral RNA and DNA in the brain, CSF, and plasma

Plasma, CSF, and brain viral RNA and DNA were quantified using an established quantitative RT-PCR (qRT-PCR) assays from the AIDS and Cancer Virus Program, Leidos Biomedical Research Inc., Frederick National Laboratory by reported methods [64] described by Li et al. [17] In brief, quantification of plasma, CSF, and brain SHIV RNA and DNA were performed using real-time qRT-PCR and qPCR assays targeting a conserved region in SIV gag. 

### Serum biochemistry

Biochemistry analysis on serum samples was performed using Piccolo® BioChemistry Plus disks, that were run on the Piccolo® Xpress Chemistry Analyzer (Abbott), according to the manufacturer’s instructions. Serum analytes that were assessed include albumin, alkaline phosphatase (ALP), amylase, aspartate aminotransferase (AST), C-reactive protein, calcium, creatinine, gamma glutamyl transferase (GGT), glucose, total protein, blood urea nitrogen (BUN), and uric acid.

### CSF and serum IP-10

A Legendplex assay (BioLegend) was performed to evaluate cytokines in rhesus macaque sera and CSF. The assay was performed according to the manufacturer’s instructions. Samples were acquired on a BD LSR Fortessa cell analyzer.

### Statistics

Differentially expressed genes (DEGs) and corresponding *p* values were determined using linear mixed effects models taking into account brain region and/or SHIV infection effects. Benjamini–Hochberg False discovery rate adjusted *p* values were utilized to account for type I errors. DEGs were defined as *p* < 0.01 and Log_2_FC > 1.5 unless otherwise noted. Two-tailed Spearman correlation analyses were used for comparing vRNA versus vDNA copy numbers and plasma versus CSF vRNA levels. Fisher’s exact test was used to identify significantly enriched GO terms within gene sets. Wilcoxon signed ranked test was used for longitudinal comparison of CSF T cell frequencies in SHIV infected animals.

## Supplementary Information

**Additional file 1: Fig. S1.** Ranked genes by median normalized read counts in units of Log_2_ counts per million (Log_2_CPM) in the subcortical white matter of the pre-frontal cortex (PFCw), and gray matter of the superior temporal sulcus (STS), caudate nucleus (CN), and hippocampus (HP) of uninfected animals. Dotted lines indicate location of marker genes associated with neurons (MAP2), astrocytes (GFAP), microglia (P2RY12), and oligodendrocytes (MOG) within ranked distribution. **Fig. S2.** T-stochastic neighborhood embedding analysis (t-SNE) of gene expression profiles from the pre-frontal cortex white matter (black), superior temporal sulcus (blue), caudate nucleus (red), and hippocampus (green) of uninfected animals. Outlier sample [Animal 43661 pre-frontal cortex white matter] is included. Symbols represent individual animals. Circles indicate 95% confidence intervals. **Fig. S3.** Regional eigengene expression and corresponding top fifteen most significantly enriched (*p* < 0.01 by Fisher’s exact test) biological processes GO terms within region specific modules (PFCw-specific [MEmagenta, MEmidnightblue], STS-specific [MEtan], CN-specific [MEpurple, MEred], HP-specific [MEsalmon]) determined by weighted gene co-expression network analysis from uninfected animals. *p < 0.05 by linear mixed effects model (region effect). Boxplots represent quartiles. **Fig. S4.** Normalized read counts of genes encoding for chemokines in the STS (blue), PFCw (black), CN (red), and HP (green) of uninfected animals. Expression levels are displayed in normalized read counts in units of Log_2_ counts per million (Log_2_CPM). Brackets indicate structural chemokine classes. **Fig. S5.** Log_2_ Fold change of genes regulating inflammatory processes and synaptic functions between SHIV infected and uninfected animals in all brain regions (gray), STS (blue), and PFCw (red). Dotted line indicates a fold change of 1**. Fig. S6.** T-stochastic neighborhood embedding (t-SNE) analysis of gene expression profiles from SHIV infected and uninfected animals. (Left) t-SNE plot indicates clustering of gene expression profiles by region and SHIV infection status (SHIV infected [pink], uninfected [black]) with removal of outlier sample [Animal 43661 Pre-frontal cortex white matter]. (Right) t-SNE plot shows all samples including the outlier with data points indicating regions (pre-frontal cortex white matter (P), superior temporal sulcus [S], caudate nucleus [C], hippocampus [H]) and infection status (color) and individual animals (symbols). Circles indicate 95% confidence intervals. **Fig. S7** Expression levels of genes (expressed as normalized read counts in units of Log_2_ Counts per million [CPM]) related to synaptic functions, endoplasmic reticulum stress, and ATP synthase subunits in the PFCw of SHIV infected (red) and uninfected (gray) animals. Violin plots indicate quartiles. *P* values determined by linear mixed effects model. **Table S1 **Animal/Sample Data. Animal information—Animal ID, Sex, Age, SHIV infection status, and medical cull rationale. Sample Information—Sample ID, Sample Code, Tissue identity, Tissue weight (mg), purified RNA absorbance ratios (A260/A280 and A260/A230), and sample RNA yield. **Table S3.** Reagents used for flow cytometric analysis.

**Additional file 2: Table S2. **Normalized Read Counts. Normalized read counts in units of Log_2_ Counts per Million (CPM). Sample IDs are listed in row 1 and correspond to Code in **Table S1**. Corresponding Gene.stable.ID and Gene names are listed in columns 1 and 2.

## Data Availability

RNA sequencing data sets analyzed in this study are deposited to Gene Expression Omnibus (GEO) database with the accession number GSE157690**.** All other data and materials are available upon reasonable request from the corresponding authors.

## References

[1] Mrdjen D (2018). High-dimensional single-cell mapping of central nervous system immune cells reveals distinct myeloid subsets in health, aging, and disease. Immunity.

[2] Korin B (2017). High-dimensional, single-cell characterization of the brain's immune compartment. Nat Neurosci.

[3] Pasciuto E (2020). Microglia require CD4 T cells to complete the fetal-to-adult transition. Cell.

[4] Carrithers MD (2002). Role of genetic background in P selectin-dependent immune surveillance of the central nervous system. J Neuroimmunol.

[5] Kivisakk P (2003). Human cerebrospinal fluid central memory CD4+ T cells: evidence for trafficking through choroid plexus and meninges via P-selectin. Proc Natl Acad Sci U S A.

[6] Ransohoff RM (2016). How neuroinflammation contributes to neurodegeneration. Science.

[7] Saloner R, Cysique LA (2017). HIV-associated neurocognitive disorders: a global perspective. J Int Neuropsychol Soc.

[8] Gott C (2017). Cognitive change trajectories in virally suppressed HIV-infected individuals indicate high prevalence of disease activity. PLoS ONE.

[9] Cardenas VA (2009). Evidence for ongoing brain injury in human immunodeficiency virus-positive patients treated with antiretroviral therapy. J Neurovirol.

[10] Price RW (2014). Evolving character of chronic central nervous system HIV infection. Semin Neurol.

[11] Oliveira MF (2017). Early antiretroviral therapy is associated with lower HIV DNA molecular diversity and lower inflammation in cerebrospinal fluid but does not prevent the establishment of compartmentalized hiv dna populations. PLoS Pathog.

[12] Dahl V (2014). Low levels of HIV-1 RNA detected in the cerebrospinal fluid after up to 10 years of suppressive therapy are associated with local immune activation. AIDS.

[13] Ragin AB (2015). Brain alterations within the first 100 days of HIV infection. Ann Clin Transl Neurol.

[14] Kieburtz K (1996). Cognitive performance and regional brain volume in human immunodeficiency virus type 1 infection. Arch Neurol.

[15] Israel SM (2019). Different roles of frontal versus striatal atrophy in HIV-associated neurocognitive disorders. Hum Brain Mapp.

[16] Grahn JA, Parkinson JA, Owen AM (2008). The cognitive functions of the caudate nucleus. Prog Neurobiol.

[17] Li H (2016). Envelope residue 375 substitutions in simian-human immunodeficiency viruses enhance CD4 binding and replication in rhesus macaques. Proc Natl Acad Sci USA.

[18] Bar KJ (2019). Simian-human immunodeficiency virus SHIVCH505 infection of rhesus macaques results in persistent viral replication and induces intestinal immunopathology. J Virol.

[19] Morrison JH, Baxter MG (2012). The ageing cortical synapse: hallmarks and implications for cognitive decline. Nat Rev Neurosci.

[20] Albright AV, Soldan SS, Gonzalez-Scarano F (2003). Pathogenesis of human immunodeficiency virus-induced neurological disease. J Neurovirol.

[21] Navia BA, Cho E-S, Petito CK, Price RW (1986). The AIDS dementia complex: II. Neuropathology. Ann Neurol..

[22] Kadharbatcha Saleem NL (2012). A combined MRI and histology atlas of the rhesus monkey brain in stereotaxic coordinates.

[23] Yin S (2020). Transcriptomic and open chromatin atlas of high-resolution anatomical regions in the rhesus macaque brain. Nat Commun.

[24] Stuart JM (2003). A gene-coexpression network for global discovery of conserved genetic modules. Science.

[25] Langfelder P, Horvath S (2007). Eigengene networks for studying the relationships between co-expression modules. BMC Syst Biol.

[26] Mittelbronn M (2001). Local distribution of microglia in the normal adult human central nervous system differs by up to one order of magnitude. Acta Neuropathol.

[27] Dubbelaar ML (2021). Transcriptional profiling of macaque microglia reveals an evolutionary preserved gene expression program. Brain Behav Immun Health.

[28] Zhao GY (2008). Expression of the transcription factor GATA3 in the postnatal mouse central nervous system. Neurosci Res.

[29] Campbell JH (2014). Anti-alpha4 antibody treatment blocks virus traffic to the brain and gut early, and stabilizes CNS injury late in infection. PLoS Pathog.

[30] Smolders J (2018). Tissue-resident memory T cells populate the human brain. Nat Commun.

[31] Sonobe Y (2009). Interleukin-25 expressed by brain capillary endothelial cells maintains blood-brain barrier function in a protein kinase Cepsilon-dependent manner. J Biol Chem.

[32] Verma A (2021). Monoclonal antibodies protect aged rhesus macaques from SARS-CoV-2-induced immune activation and neuroinflammation. Cell Rep.

[33] Schwerk C (2015). The choroid plexus-a multi-role player during infectious diseases of the CNS. Front Cell Neurosci.

[34] Shimada A, Hasegawa-Ishii S (2021). Increased cytokine expression in the choroid plexus stroma and epithelium in response to endotoxin-induced systemic inflammation in mice. Toxicol Rep.

[35] Baruch K (2013). CNS-specific immunity at the choroid plexus shifts toward destructive Th2 inflammation in brain aging. Proc Natl Acad Sci U S A.

[36] Sedmak G, Judas M (2021). White matter interstitial neurons in the adult human brain: 3% of cortical neurons in quest for recognition. Cells.

[37] Kostovic I, Rakic P (1980). Cytology and time of origin of interstitial neurons in the white matter in infant and adult human and monkey telencephalon. J Neurocytol.

[38] Dai R (2020). EGR2 is elevated and positively regulates inflammatory IFNgamma production in lupus CD4(+) T cells. BMC Immunol.

[39] Lin CC (2016). IL-1-induced Bhlhe40 identifies pathogenic T helper cells in a model of autoimmune neuroinflammation. J Exp Med.

[40] Valle-Rios R (2014). Isthmin 1 is a secreted protein expressed in skin, mucosal tissues, and NK, NKT, and th17 cells. J Interferon Cytokine Res.

[41] Ghosh S (2018). Bioenergetic regulation of microglia. Glia.

[42] Durocher M (2019). Inflammatory, regulatory, and autophagy co-expression modules and hub genes underlie the peripheral immune response to human intracerebral hemorrhage. J Neuroinflammation.

[43] Mengozzi M (2012). Erythropoietin-induced changes in brain gene expression reveal induction of synaptic plasticity genes in experimental stroke. Proc Natl Acad Sci U S A.

[44] Lin CC (2014). Bhlhe40 controls cytokine production by T cells and is essential for pathogenicity in autoimmune neuroinflammation. Nat Commun.

[45] Bonham LW (2018). CXCR4 involvement in neurodegenerative diseases. Transl Psychiatry.

[46] Li J (2005). IL-27 subunits and its receptor (WSX-1) mRNAs are markedly up-regulated in inflammatory cells in the CNS during experimental autoimmune encephalomyelitis. J Neurol Sci.

[47] Nitsch L (2019). CNS-specific synthesis of interleukin 23 induces a progressive cerebellar ataxia and the accumulation of both T and B cells in the brain: characterization of a novel transgenic mouse model. Mol Neurobiol.

[48] Yan L, Chaqour B (2013). Cysteine-rich protein 61 (CCN1) and connective tissue growth factor (CCN2) at the crosshairs of ocular neovascular and fibrovascular disease therapy. J Cell Commun Signal.

[49] Sun J (2020). Zika virus promotes CCN1 expression via the CaMKIIalpha-CREB pathway in astrocytes. Virulence.

[50] Rothe T (2017). The nuclear receptor Nr4a1 acts as a microglia rheostat and serves as a therapeutic target in autoimmune-driven central nervous system inflammation. J Immunol.

[51] Kessing CF (2017). High number of activated CD8+ T cells targeting HIV antigens are present in cerebrospinal fluid in acute HIV infection. J Acquir Immune Defic Syndr.

[52] Boisvert MM (2018). The aging astrocyte transcriptome from multiple regions of the mouse brain. Cell Rep.

[53] Xu S (2019). Mesencephalic astrocyte-derived neurotrophic factor (MANF) protects against Abeta toxicity via attenuating Abeta-induced endoplasmic reticulum stress. J Neuroinflammation.

[54] Lee CA (2020). Simian immunodeficiency virus-infected memory CD4(+) T cells infiltrate to the site of infected macrophages in the neuroparenchyma of a chronic macaque model of neurological complications of AIDS. MBio.

[55] Hsu DC (2018). Central nervous system inflammation and infection during early, nonaccelerated simian-human immunodeficiency virus infection in rhesus macaques. J Virol.

[56] Verma A (2020). Impact of Th1 CD4 follicular helper t cell skewing on antibody responses to an HIV-1 vaccine in rhesus macaques. J Virol.

[57] Peters A, Sethares C (2002). Aging and the myelinated fibers in prefrontal cortex and corpus callosum of the monkey. J Comp Neurol.

[58] McMurtray A (2008). Cortical atrophy and white matter hyperintensities in HIV: the Hawaii Aging with HIV Cohort Study. J Stroke Cerebrovasc Dis.

[59] Cohen RA, Seider TR, Navia B (2015). HIV effects on age-associated neurocognitive dysfunction: premature cognitive aging or neurodegenerative disease?. Alzheimers Res Ther.

[63] Bowen L, von Biela VR, McCormick SD, Regish AM, Waters SC, Durbin-Johnson B, Britton M, Settles ML, Donnelly DS, Laske SM, Carey MP, Brown RJ, Zimmerman CE, Cooke S (2020). Transcriptomic response to elevated water temperatures in adult migrating Yukon River Chinook salmon (Oncorhynchus tshawytscha). Conserv Physiol..

[61] Dobin A (2012). STAR: ultrafast universal RNA-seq aligner. Bioinformatics.

[62] Ge SX, Jung D, Yao R (2019). ShinyGO: a graphical gene-set enrichment tool for animals and plants. Bioinformatics.

[63] Assarsson E (2014). Homogenous 96-plex PEA immunoassay exhibiting high sensitivity, specificity, and excellent scalability. PLoS ONE.

[64] Hansen SG (2013). Immune clearance of highly pathogenic SIV infection. Nature.

