# Supplementary material for: Neuroinflammatory transcriptional programs induced in rhesus pre-frontal cortex white matter during acute SHIV infection
Source: J Neuroinflammation. 2022 Oct 6;19:250. doi: 10.1186/s12974-022-02610-y (PMC9535930; doi:10.1186/s12974-022-02610-y)
Supplement: Supplementary file 1 — Additional file 1: Fig. S1. Ranked genes by median normalized read counts in units of Log2 counts per million (Log2CPM) in the subcortical white matter of the pre-frontal cortex (PFCw), and gray matter of the superior temporal sulcus (STS), caudate nucleus (CN), and hippocampus (HP) of uninfected animals. Dotted lines indicate location of marker genes associated with neurons (MAP2), astrocytes (GFAP), microglia (P2RY12), and oligodendrocytes (MOG) within ranked distribution. Fig. S2. T-stochastic neighborhood embedding analysis (t-SNE) of gene expression profiles from the pre-frontal cortex white matter (black), superior temporal sulcus (blue), caudate nucleus (red), and hippocampus (green) of uninfected animals. Outlier sample [Animal 43661 pre-frontal cortex white matter] is included. Symbols represent individual animals. Circles indicate 95% confidence intervals. Fig. S3. Regional eigengene expression and corresponding top fifteen most significantly enriched (p < 0.01 by Fisher’s exact test) biological processes GO terms within region specific modules (PFCw-specific [MEmagenta, MEmidnightblue], STS-specific [MEtan], CN-specific [MEpurple, MEred], HP-specific [MEsalmon]) determined by weighted gene co-expression network analysis from uninfected animals. *p < 0.05 by linear mixed effects model (region effect). Boxplots represent quartiles. Fig. S4. Normalized read counts of genes encoding for chemokines in the STS (blue), PFCw (black), CN (red), and HP (green) of uninfected animals. Expression levels are displayed in normalized read counts in units of Log2 counts per million (Log2CPM). Brackets indicate structural chemokine classes. Fig. S5. Log2 Fold change of genes regulating inflammatory processes and synaptic functions between SHIV infected and uninfected animals in all brain regions (gray), STS (blue), and PFCw (red). Dotted line indicates a fold change of 1. Fig. S6. T-stochastic neighborhood embedding (t-SNE) analysis of gene expression profiles from SHIV in [file 12974_2022_2610_MOESM1_ESM.pdf]

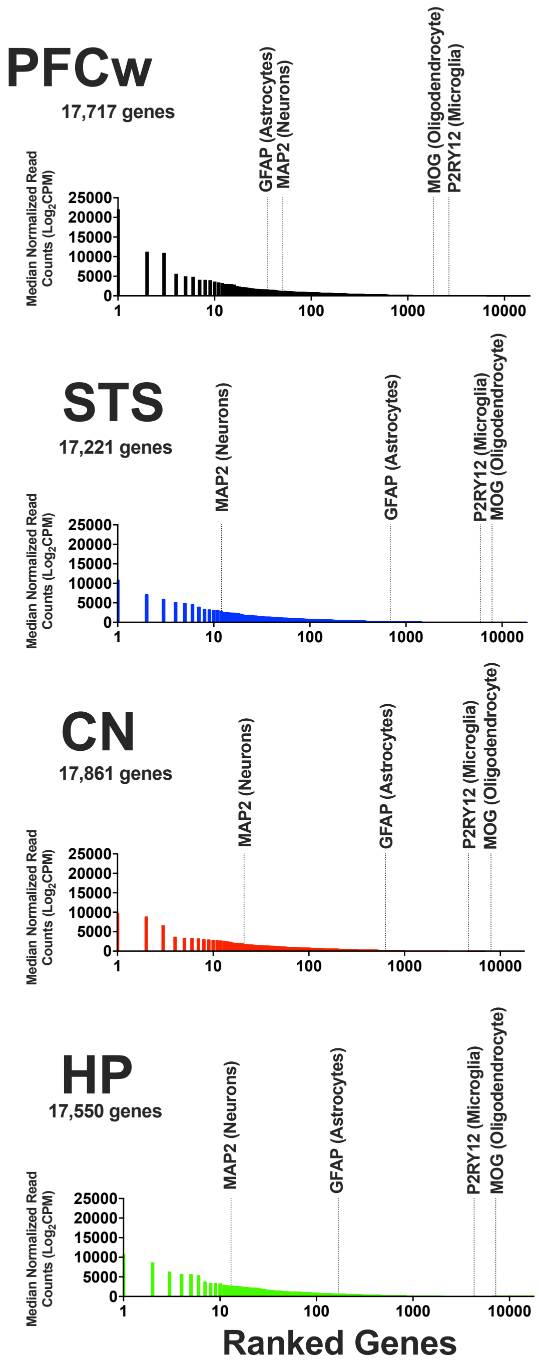
PFCw
17,717 genes
GFAP (Astrocytes)
MAP2 (Neurons)
MOG (Oligodendrocyte)
P2RY12 (Microglia)
STS
17,221 genes
MAP2 (Neurons)
GFAP (Astrocytes)
P2RY12 (Microglia)
MOG (Oligodendrocyte)
CN
17,861 genes
MAP2 (Neurons)
GFAP (Astrocytes)
P2RY12 (Microglia)
MOG (Oligodendrocyte)
HP
17,550 genes
MAP2 (Neurons)
GFAP (Astrocytes)
P2RY12 (Microglia)
MOG (Oligodendrocyte)
Median Normalized Read Counts (Log_2CPM)
25000
20000
15000
10000
5000
0
1
10
100
1000
10000
Ranked Genes

S1

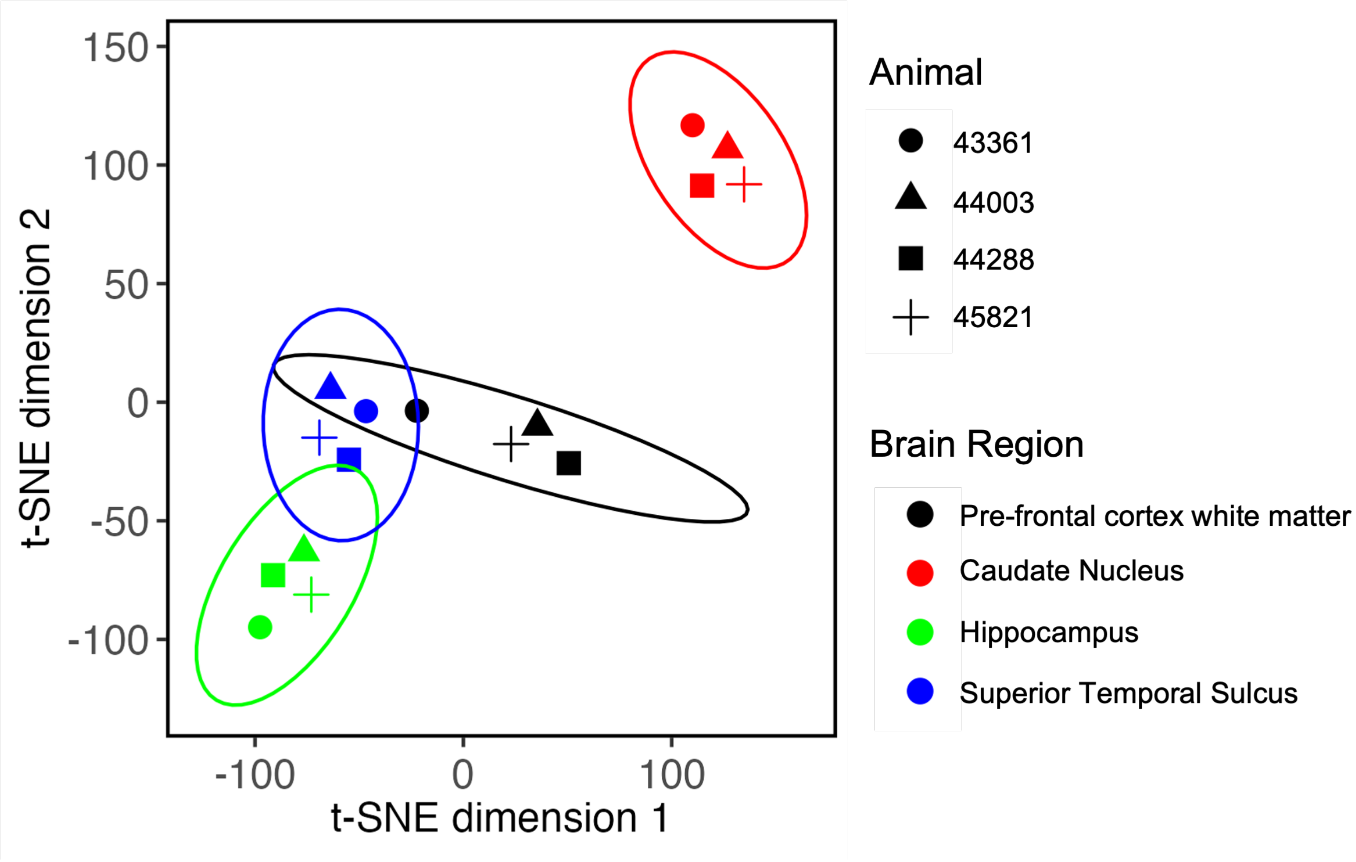
150
100
50
0
-50
-100
t-SNE dimension 2
-100
0
100
t-SNE dimension 1
Animal
43361
44003
44288
45821
Brain Region
Pre-frontal cortex white matter
Caudate Nucleus
Hippocampus
Superior Temporal Sulcus

S2

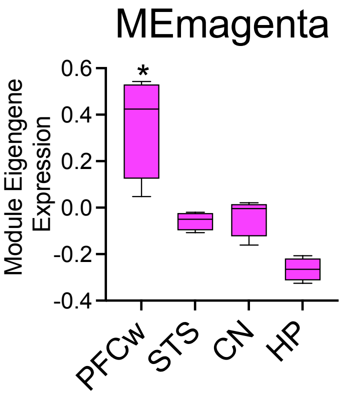

- cellular response to glucocorticoid stimulus
- nucleosome mobilization
- RNA metabolic process
- microvillus organization
- response to antineoplastic agent
- negative regulation of cellular component organization
- amyloid-beta clearance
- regulation of extrinsic apoptotic signaling pathway via death domain receptors
- dendritic transport
- axo-dendritic protein transport
- negative regulation of extrinsic apoptotic signaling pathway
- regulation of RNA metabolic process
- apical junction assembly
- regulation of macromolecule biosynthetic process
- response to arsenic-containing substance

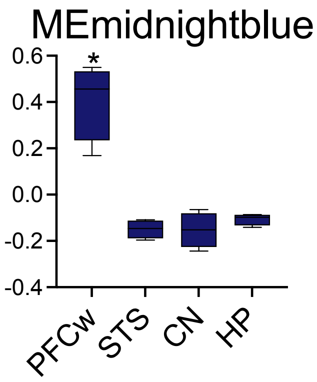

- positive regulation of dendritic spine development
- N-glycan fucosylation
- positive regulation of L-glutamate import across plasma membrane
- regulation of dendritic spine maintenance
- arsonoacetate metabolic process
- activation of cysteine-type endopeptidase activity involved in apoptotic process by cytochrome c
- negative regulation of core promoter binding
- Cajal-Retzius cell differentiation
- regulation of granulosa cell apoptotic process
- negative regulation of nucleotide-binding oligomerization domain containing 2 signaling pathway
- smooth endoplasmic reticulum calcium ion homeostasis
- positive regulation of amyloid fibril formation
- negative regulation of monocyte chemotactic protein-1 production
- negative regulation of mitochondrial depolarization
- synaptic vesicle targeting

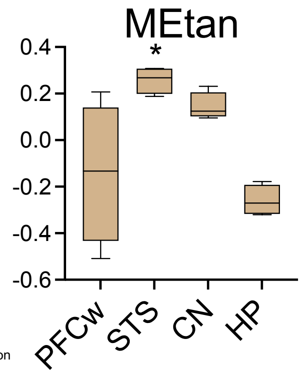

- mechanosensory behavior
- cation transport
- neuron projection arborization
- regulation of ketone biosynthetic process
- regulation of cholesterol metabolic process
- calcium ion-regulated exocytosis of neurotransmitter
- cellular response to calcium ion
- release of sequestered calcium ion into cytosol
- metal ion transport
- neutral lipid metabolic process
- acylglycerol metabolic process
- rhythmic behavior
- circadian behavior
- circadian temperature homeostasis
- protein demalonylation

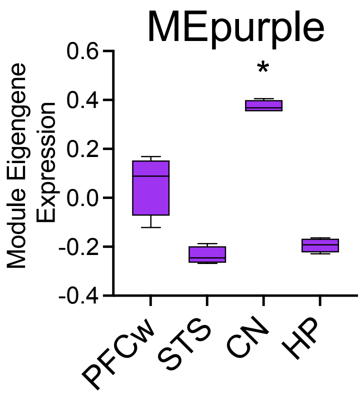

- spermatogenesis
- DNA methylation involved in gamete generation
- auditory receptor cell morphogenesis
- cochlea development
- neural precursor cell proliferation
- stem cell proliferation
- cellular response to amino acid stimulus
- cellular response to organonitrogen compound
- insulin receptor signaling pathway
- positive regulation of mRNA splicing, via spliceosome
- regulation of neural precursor cell proliferation
- response to amino acid
- regulation of cell shape
- mucopolysaccharide metabolic process
- eye development

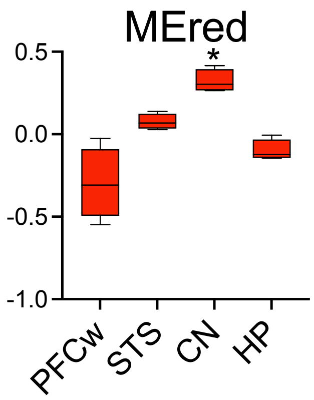

- modulation of chemical synaptic transmission
- neutrophil extravasation
- postsynaptic neurotransmitter receptor diffusion trapping
- monocyte extravasation
- calcium ion transport
- inorganic cation transmembrane transport
- cellular response to hormone stimulus
- heme biosynthetic process
- ATP metabolic process
- neurotransmitter transport
- cellular response to glucose stimulus
- cellular respiration
- adenylate cyclase-activating dopamine receptor signaling pathway
- negative regulation of transcription by RNA polymerase III
- presynaptic modulation of chemical synaptic transmission

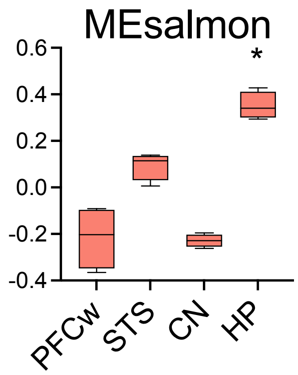

- psychomotor behavior
- social behavior
- neuron projection development
- synaptic vesicle priming
- regulation of cell morphogenesis involved in differentiation
- microtubule polymerization
- positive regulation of protein modification by small protein conjugation or removal
- long-term memory
- regulation of microtubule polymerization or depolymerization
- negative regulation of endothelial cell proliferation
- positive regulation of substrate adhesion-dependent cell spreading
- positive regulation of transferase activity
- synaptic vesicle lumen acidification
- cerebral cortex GABAergic interneuron development
- nonassociative learning

S3

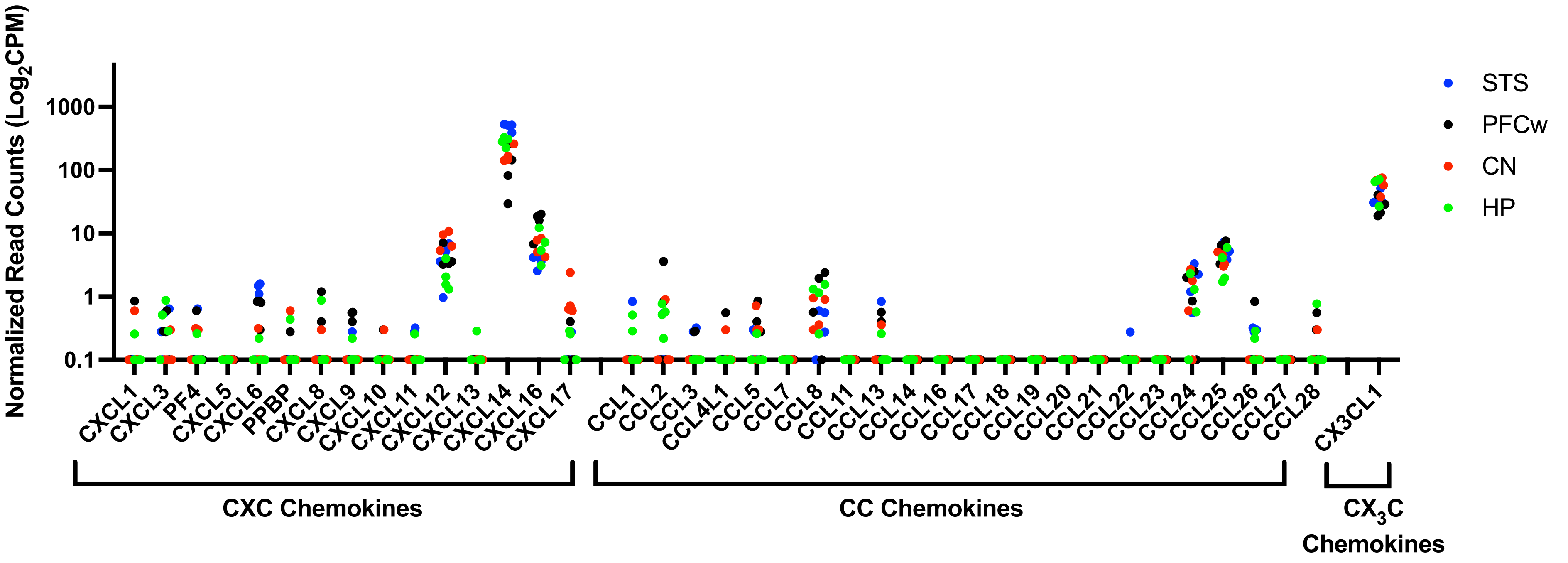

Normalized Read Counts (Log2CPM)
1000
100
10
1
0.1
STS
PFCw
CN
HP
CXCL1
CXCL3
PF4
CXCL5
CXCL6
PPBP
CXCL8
CXCL9
CXCL10
CXCL11
CXCL12
CXCL13
CXCL14
CXCL16
CXCL17
CCL1
CCL2
CCL3
CCL4L1
CCL5
CCL7
CCL8
CCL11
CCL13
CCL14
CCL16
CCL17
CCL18
CCL19
CCL20
CCL21
CCL22
CCL23
CCL24
CCL25
CCL26
CCL27
CCL28
CX3CL1
CXC Chemokines
CC Chemokines
CX3C Chemokines

S4

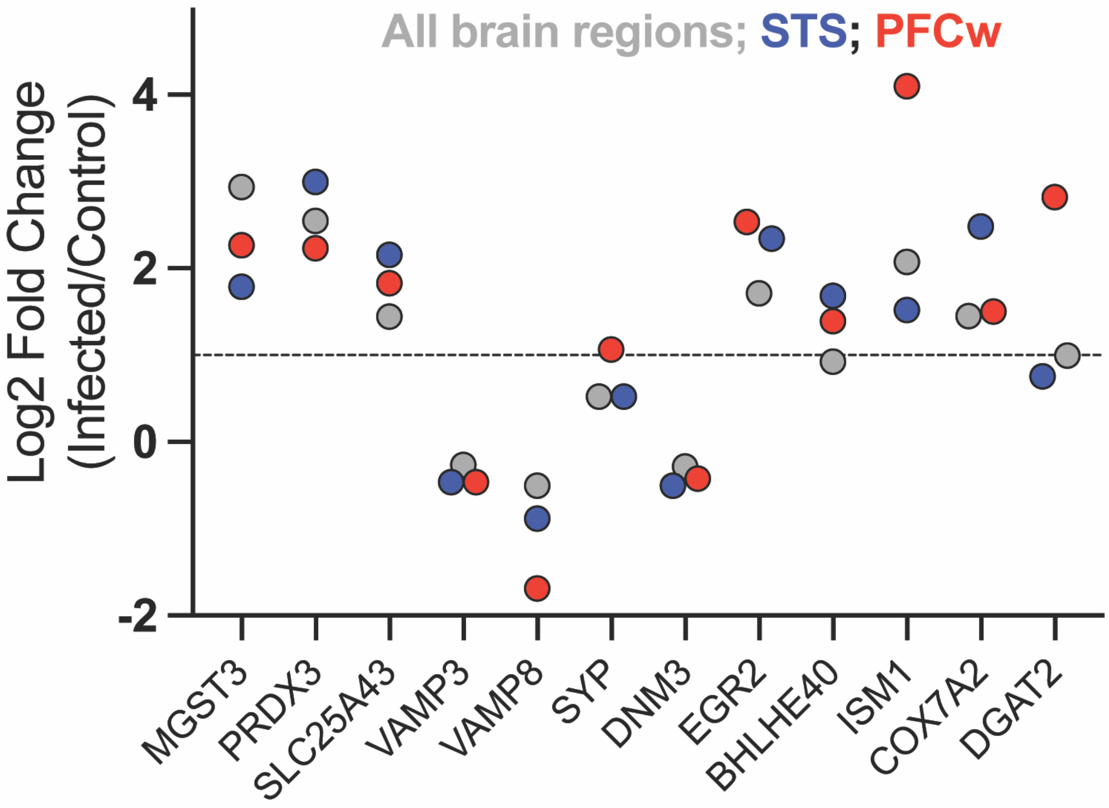
All brain regions; STS; PFCw
Log2 Fold Change
(Infected/Control)
4
2
0
-2
MGST3
PRDX3
SLC25A43
VAMP3
VAMP8
SYP
DNM3
EGR2
BHLHE40
ISM1
COX7A2
DGAT2

S5

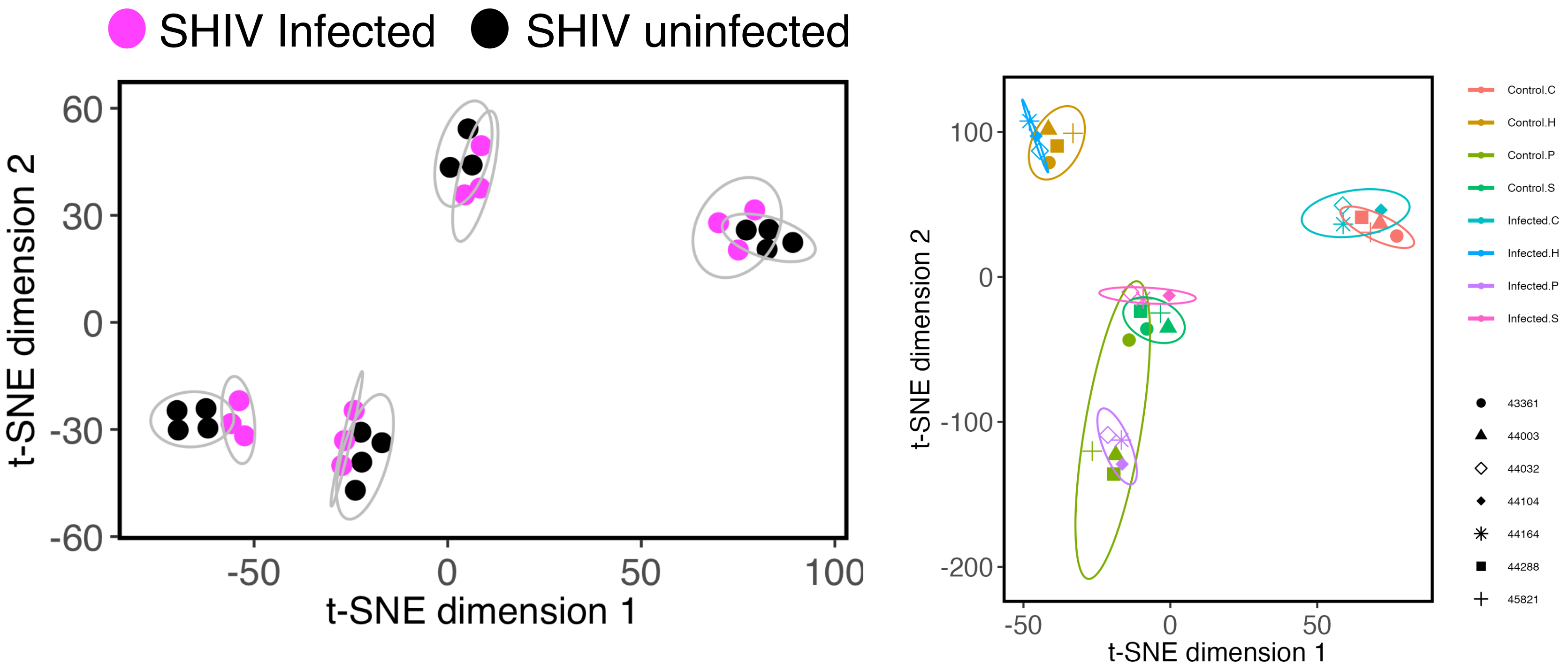
SHIV Infected
SHIV uninfected
t-SNE dimension 2
60
30
0
-30
-60
-50
0
50
100
t-SNE dimension 1
t-SNE dimension 2
100
0
-100
-200
-50
0
50
t-SNE dimension 1
Control.C
Control.H
Control.P
Control.S
Infected.C
Infected.H
Infected.P
Infected.S
43361
44003
44032
44104
44164
44288
45821

S6

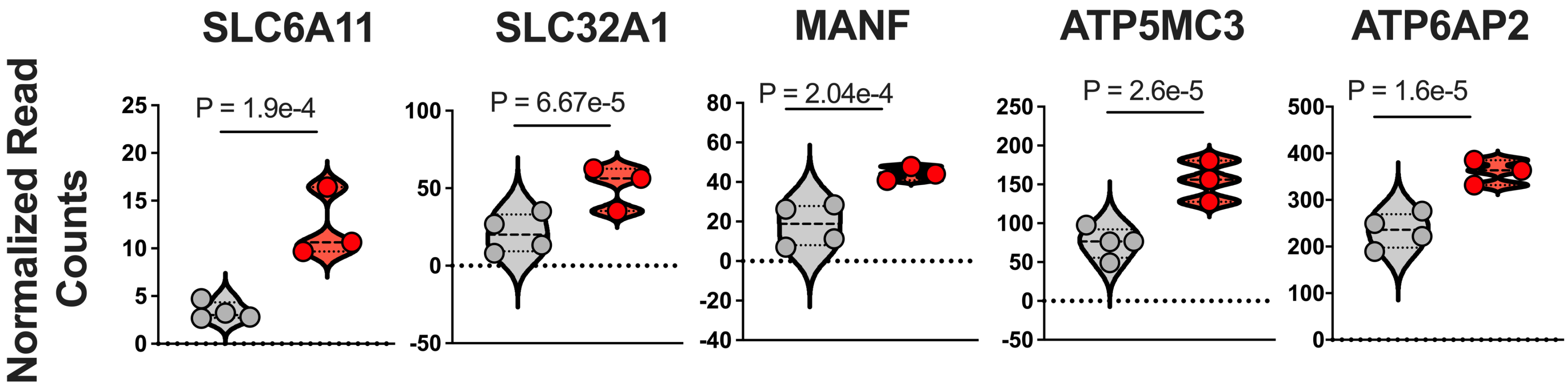
Normalized Read Counts
SLC6A11
P = 1.9e-4
0
5
10
15
20
25
SLC32A1
P = 6.67e-5
-50
0
50
100
MANF
P = 2.04e-4
-40
-20
0
20
40
60
80
ATP5MC3
P = 2.6e-5
-50
0
50
100
150
200
250
ATP6AP2
P = 1.6e-5
0
100
200
300
400
500

S7

Table S1. Animal/Sample Data

Animal ID	Sample ID	SHIV Exposed	Sex	Age (years.months)	Med cull	Code	Brain tissue	Tissue weight (mg)	A260/A280	A260/A230	Total RNA conc (ug)
43361	SI61	No	M	6.6	Right leg lameness	SI61S-1	Superior Temporal Sulcus	74.0	2.06	2.24	25.74
						SI61H-2	Hippocampus	114.2	2.08	2.27	39.16
						SI61C-3	Caudate Nucleus	91.6	2.09	2.22	33.06
						SI61P-4	PFC A46 (w)	53.5	2.07	2.27	22.96
45821	SI21	No	F	3.6	Arthritis	SI21S-5	Superior Temporal Sulcus	124.8	2.10	2.30	53.59
						SI21H-6	Hippocampus	50.5	2.10	2.25	31.2
						SI21C-7	Caudate Nucleus	65.6	2.11	2.28	34.23
						SI21P-8	PFC A46 (w)	39.5	2.10	2.07	15.3
44003	SI03	No	F	5. 11	Arthritis	SI03S-9	Superior Temporal Sulcus	84.2	2.10	2.31	50.42
						SI03H-10	Hippocampus	76.7	2.09	1.86	30.45
						SI03C-11	Caudate Nucleus	90.1	2.09	1.96	32.4
						SI03P-12	PFC A46 (w)	72.3	2.05	2.13	16.8
44288	SI88	No	F	5.9	Diarrhea	SI88S-13	Superior Temporal Sulcus	104.2	2.07	2.27	33.58
						SI88H-14	Hippocampus	83.9	2.07	2.28	29.11
						SI88C-15	Caudate Nucleus	104.5	2.08	2.25	31.01
						SI88P-16	PFC A46 (w)	69.8	2.06	2.15	9.89
44164	SI64	Yes	F	5.5	Euthanasia 4 weeks post SHIV	SI64S-17	Superior Temporal Sulcus	40.2	2.05	2.12	19.8
						SI64H-18	Hippocampus	50.4	2.07	2.20	27.57
						SI64C-19	Caudate Nucleus	35.9	2.06	1.89	20.32
						SI64P-20	PFC A46 (w)	26.7	2.07	2.22	14.45
44104	SI04	Yes	F	5.2	Euthanasia 4 weeks post SHIV	SI04S-21	Superior Temporal Sulcus	52.3	2.08	2.30	29.22
						SI04H-22	Hippocampus	43.3	2.06	2.29	21.26
						SI04C-23	Caudate Nucleus	47.2	2.07	2.37	21.64
						SI04P-24	PFC A46 (w)	17.0	2.08	2.18	9.6
44032	SI25	Yes	F	5.6	Euthanasia 4 weeks post SHIV	SI32S-25	Superior Temporal Sulcus	46.5	2.07	2.24	21.56
						SI32H-26	Hippocampus	54.3	2.06	2.20	23.57
						SI32C-27	Caudate Nucleus	40.6	2.08	2.22	19.22
						SI32P-28	PFC A46 (w)	43.5	2.06	2.21	20.06

Table S3 Flow Cytometry Reagents

Reagent	Flurochrome	Clone	Catalog no.	Source
**PD-1**	PE-Cy7	EH12.2H7	329918	Biolegend
**CD3**	AF700	SP34-2	557917	BD Biosciences
**CD4**	BV650	L200	563737	BD Biosciences
**CD8**	BV510	SK1	563919	BD Biosciences
**CD20**	BV421	2H7	302328	Biolegend
**CD95**	BUV 737	DX2	564710	BD Bioscience
**Live/Dead**	APC-Cy7		L34976	Life Technologies
**CXCR3**	APC	IC6	550967	BD Biosciences
**CD45**	AF-488	HI30	557803	BD Biosciences
**CD11b**	BV-510	ICRF44	563088	Thermofisher
**HLA-DR**	BV-785	L243	307642	Biolegend
**CCR6**	PE-Dazzle	G034e3	353430	Biolegend
**Cytofix/cytoperm**			51-2090KZ	BD Biosciences
